# Transcriptome profile of spleen tissues from locally-adapted Kenyan pigs (*Sus scrofa)* experimentally infected with three varying doses of a highly virulent African swine fever virus genotype IX isolate: Ken12/busia.1 (ken-1033)

**DOI:** 10.1186/s12864-022-08754-8

**Published:** 2022-07-19

**Authors:** Eunice Magoma Machuka, John Juma, Anne Wangari Thairu Muigai, Joshua Oluoch Amimo, Roger Pelle, Edward Okoth Abworo

**Affiliations:** 1 Animal and Human Health Program, International Livestock Research Institute (ILRI), P.O. Box 30709-00100, Nairobi, Kenya; 2 Pan African University Institute for Basic Sciences Technology and Innovation (PAUSTI), P.O Box 62000-00200, Nairobi, Kenya; 3 Botany Department, Jomo Kenyatta University of Agriculture and Technology, P.O Box, Juja, Kenya; 4 Center for Food Animal Health, Department of Animal Sciences, The Ohio State University, 1680 Madison Avenue, Wooster, OH 44691 USA; 5 Biosciences eastern and central Africa, International Livestock Research Institute (BecA-ILRI) Hub, P.O. Box 30709-00100, Nairobi, Kenya

**Keywords:** Dual RNA-Seq, Swine, Locally-adapted pigs, ASFV, Vaccine, Cytokines, Chemokines, Spleen

## Abstract

**Background:**

African swine fever (ASF) is a lethal hemorrhagic disease affecting domestic pigs resulting in up to 100% mortality rates caused by the ASF virus (ASFV). The locally-adapted pigs in South-western Kenya have been reported to be resilient to disease and harsh climatic conditions and tolerate ASF; however, the mechanisms by which this tolerance is sustained remain largely unknown. We evaluated the gene expression patterns in spleen tissues of these locally-adapted pigs in response to varying infective doses of ASFV to elucidate the virus-host interaction dynamics.

**Methods:**

Locally adapted pigs (*n* = 14) were experimentally infected with a high dose (1x10^6^HAD_50_), medium dose (1x10^4^HAD_50_), and low dose (1x10^2^HAD_50_) of the highly virulent genotype IX ASFV Ken12/busia.1 (Ken-1033) isolate diluted in PBS and followed through the course of infection for 29 days. The in vivo pig host and ASFV pathogen gene expression in spleen tissues from 10 pigs (including three from each infective group and one uninfected control) were analyzed in a dual-RNASeq fashion. We compared gene expression between three varying doses in the host and pathogen by contrasting experiment groups against the naïve control.

**Results:**

A total of 4954 differentially expressed genes (DEGs) were detected after ASFV Ken12/1 infection, including 3055, 1771, and 128 DEGs in the high, medium, and low doses, respectively. Gene ontology and KEGG pathway analysis showed that the DEGs were enriched for genes involved in the innate immune response, inflammatory response, autophagy, and apoptosis in lethal dose groups. The surviving low dose group suppressed genes in pathways of physiopathological importance. We found a strong association between severe ASF pathogenesis in the high and medium dose groups with upregulation of proinflammatory cytokines and immunomodulation of cytokine expression possibly induced by overproduction of prostaglandin E synthase (4-fold; *p* < 0.05) or through downregulation of expression of M1-activating receptors, signal transductors, and transcription factors. The host-pathogen interaction resulted in induction of expression of immune-suppressive cytokines (*IL-27*), inactivation of autophagy and apoptosis through up-regulation of *NUPR1* [5.7-fold (high dose) and 5.1-fold (medium dose) [*p* < 0.05] and IL7R expression. We detected repression of genes involved in MHC class II antigen processing and presentation, such as cathepsins, *SLA-DQB1, SLA-DOB, SLA-DMB, SLA-DRA,* and *SLA-DQA* in the medium and high dose groups. Additionally, the host-pathogen interaction activated the CD8^+^ cytotoxicity and neutrophil machinery by increasing the expression of neutrophils/CD8^+^ T effector cell-recruiting chemokines (*CCL2, CXCL2, CXCL10, CCL23, CCL4, CXCL8,* and *CXCL13*) in the lethal high and medium dose groups. The recovered pigs infected with ASFV at a low dose significantly repressed the expression of *CXCL10*, averting induction of T lymphocyte apoptosis and *FUNDC1* that suppressed neutrophilia.

**Conclusions:**

We provide the first in vivo gene expression profile data from locally-adapted pigs from south-western Kenya following experimental infection with a highly virulent ASFV genotype IX isolate at varying doses that mimic acute and mild disease. Our study showed that the locally-adapted pigs induced the expression of genes associated with tolerance to infection and repression of genes involved in inflammation at varying levels depending upon the ASFV dose administered.

**Supplementary Information:**

The online version contains supplementary material available at 10.1186/s12864-022-08754-8.

## Introduction

African swine fever (ASF) is an important notifiable transboundary disease that impedes the pig value chain and threatens global pig productivity and food security [1]. The aetiologic agent is the African swine fever virus (ASFV), a large double-stranded DNA virus. ASFV was first reported in Kenya in 1921 [2] and has continued to spread to Europe and Asia, causing global food security concerns. ASF is a contagious disease transmitted by direct contact between infected pigs and susceptible ones, through contact with infectious secretions and excretions, fomites [3], or tick vectors *Ornithodoros* spp. [4–6]. ASFV persists in tissues [7] and the environment, transmitting over long distances through swill containing pork and pork products and fomites such as contaminated material, vehicles, or visitors to pig premises [3]. ASF causes persistent outbreaks in endemic and non-endemic regions [8]. Currently, no effective vaccine or treatment is available and the control measures entail strict biosecurity measures like slaughtering infected and exposed animals (stamping out), movement and trade restrictions [9–13]. Ongoing efforts on ASF vaccine design focus on targeted gene deletion to attenuate virulent viruses or develop subunit vaccines targeting known protective antigens. However, these face a bottleneck in optimal delivery systems, limited knowledge of protective antigens [14], and a high risk of converting the attenuated virus to a virulent strain [11].

Presently, ASFV is the only member of the family *Asfarviridae*, genus *Asfivirus* [15], whose genome is a double-stranded DNA of about 170–195 kb that encodes 150–195 genes that code for viral proteins responsible for normal cellular metabolic activity, DNA replication, repair, and modulation of host immunity and multigene families [16]. Replication of ASFV occurs in (i) the swine macrophages and monocytes, where it causes an overproduction of cytokines leading to induction of apoptosis in pig cells [9] and (ii) soft ticks (*Ornithodoros* spp.) in which the virus replicates and is transmitted to other susceptible swine hosts following tick bites [17]. In domestic pigs, clinical manifestations vary depending on the virus strain. Virulent ASF virus strains result in 100% mortality. The acute form of ASF develops over seven days, compared with 10–20 days for the sub-acute form of the disease in which the virus is shed up to 70 days from the oropharynx [18]. The varying clinical forms pose a challenge in diagnosis and persist in surviving pigs, which may serve as reservoirs for future outbreaks [19]. The moderate-to-low virulence ASF virus isolates trigger persistent viral infections, resulting in ASF persistence in endemic areas [1, 20]. Since ASFV primarily affects cells of the mononuclear phagocytic system [21], the interaction between ASFV and the host macrophages thus influences the pathognomonic outcome [22]. Animals with acute ASF display fever and a tendency to crowd, loss of appetite, inactivity, apathy and early leukopenia induced by lymphopenia and changes in monocyte numbers [23]. Affected pigs show erythema mainly affecting the skin of the ears, tail, distal extremities, chest, abdomen and perianal area, and vascular lesions and cyanosis of the skin tend to be more apparent in exotic pig breeds. Other symptoms include vomiting, abdominal pain, constipation, and diarrhoea that was initially mucoid but may later become bloody [24].

The fact that ASFV can persist in its natural hosts comprising warthogs, bush pigs, soft ticks and recovered domestic pigs, confirmed that the virus has effective mechanisms to evade detection by the host defense system [6, 25]. The key pathway modulated by the virus to evade host immunity is the macrophage signaling pathway resulting in altered expression of the immunomodulatory genes and the calcineurin-dependent pathways [6, 25, 26]. Studies have shown that some pigs are very susceptible to ASF, while others are asymptomatic carriers of the virus, despite infection with similar virus genotypes [7, 25, 27, 28]. The locally-adapted pigs are favorites among smallholder farmers in many parts of the world [29, 30], including in South-western Kenya, where ASFV persistence in tissues of apparently healthy pigs has been reported [7]. The level of protection has been attributed to antibody-dependent cytotoxicity that offers limited protection only against homologous ASFV challenge, delaying the onset of clinical signs and viremia [31–33]. A recent study by Franzoni and colleagues showed that virulent ASFV isolates had evolved mechanisms to upset activated macrophage response, promoting viral survival and dissemination in the host and pathogenesis [34]. To date, numerous viral proteins have been identified as immunogenic [35], but the mechanisms by which they elicit an effective immune response in surviving animals remain poorly described. Understanding how ASFV persists in hosts is needed to design better therapeutic strategies [6, 36]. But gaps still exist in the characterization of the host-pathogen interactions, which may yield new insights into how to induce a protective immune response.

RNA-Seq is now extensively applied in differential expression studies yielding vast amounts of data matched to a reference genome [37–39]. In pigs, RNA-Seq studies have compared breeds for development and meat quality using brain, liver, and tonsil tissues [40–43] and immunological determination in tonsils, blood and lymph nodes [40, 44, 45]. There are minimal transcriptomic studies that assess other pig organs, such as the spleen [46], with none hitherto assessing pig spleen gene expression following ASFV infection. The spleen tissue is a secondary lymphoid organ with various immunologic functions alongside hematopoiesis and clearance of red blood cells [47, 48]. Its structure allows it to filter blood containing pathogens and abnormal cells with a high prospect of interacting with antigen-presenting cells (APCs) and related lymphocytes [47, 48].

The current study aimed to determine the in vivo host and pathogen gene expression profile in spleen tissues of locally-adapted Kenyan pigs, following experimental challenge with varying (low, medium, and high) infective doses of a highly virulent ASFV isolate, the Ken12/busia.1 responsible for ASF outbreaks in East Africa. The ASFV Ken12/busia.1 isolate was originally isolated from the spleen of a pig sacrificed in the former Busia district (now Busia County) in Western Kenya during a longitudinal survey of ASF and has been confirmed to belong to Genotype IX [49]. The gene expression profiles between the infective dose groups show common and unique patterns between the medium and high dose groups than with the low dose groups with upregulation of pig host genes associated with macrophages, NK cells, and viral genes associated with modification of host immunity.

## Results

### Clinical outcomes and pathology

**Fig. 1:**
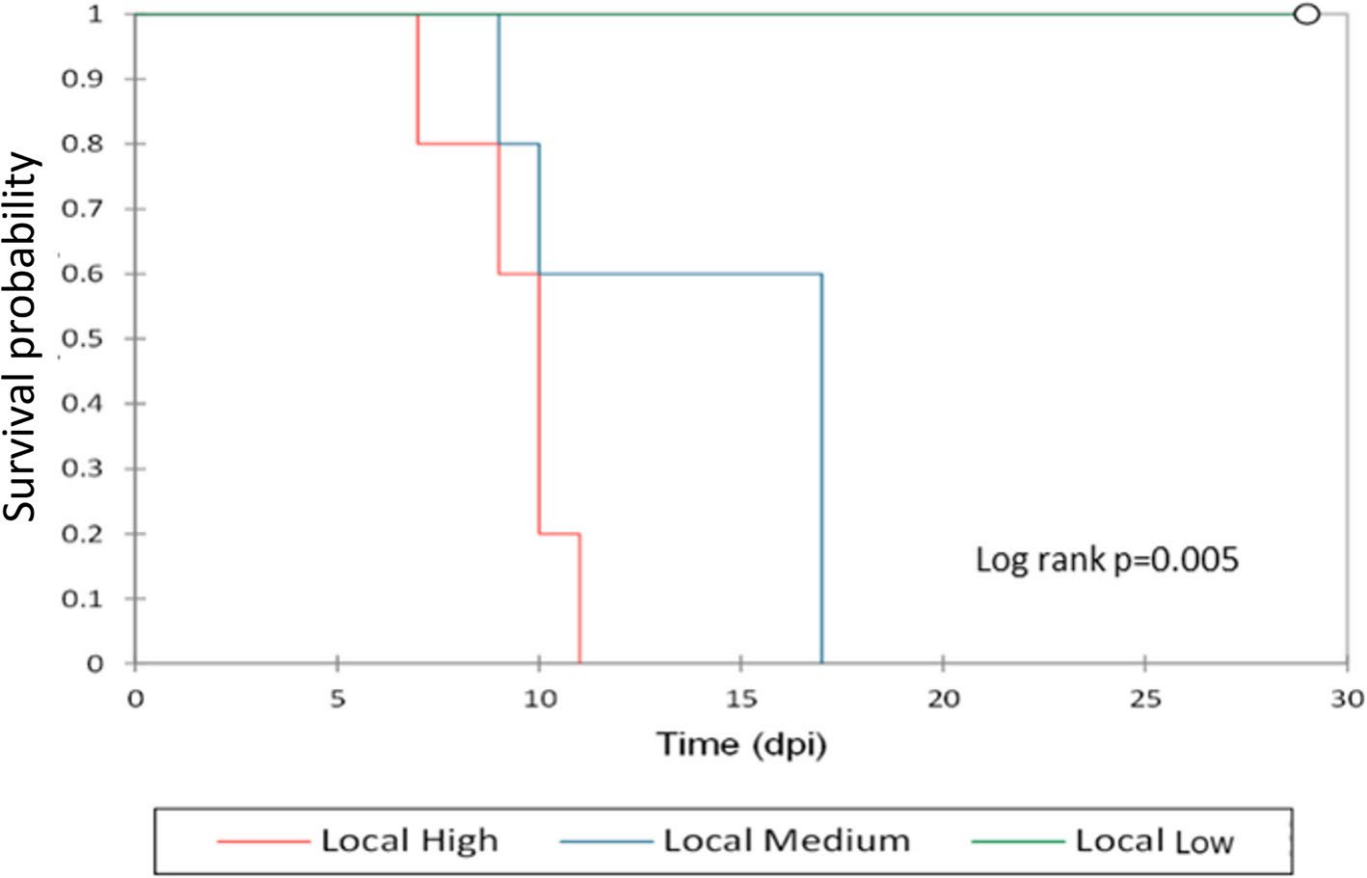
A survival analysis curve

The clinical signs recorded were intermittent fever, high body temperature (> 40 °C), depression, and anorexia. The pigs in the low dose group did not show any ASF clinical signs or gross pathology throughout the experiment and were euthanized at the termination of the experiment (29 dpi: Fig. 1). From 7 dpi, all locally-adapted pigs in the medium and high dose groups showed acute ASF clinical signs, including depression, anorexia, recumbence, accelerated and labored breathing, diarrhea, and slight ataxia, and were euthanized humanely from 7 dpi (*n* = 1 in high dose), 9 dpi (*n* = 2; 1 each from high and medium dose), 10 dpi (*n* = 3; 2 from high dose and 1 from medium dose), 11 dpi (*n* = 1 from medium dose) and 17 dpi (*n* = 3 from medium dose). The locally-adapted pigs have black pigment, and as a result, skin lesions could not be scored. We also observed recumbence, reduced feed intake, weight loss from 4 dpi, and foul-smelling watery diarrhea. The highest temperature (39.6 °C) was recorded at 7 dpi from the first pig that succumbed at 7 dpi from the high dose group (Supplementary Table 1 and Fig. 1- survival analysis). From 9 dpi, all the remaining locally-adapted pigs showed febrile temperature reactions (40.5 °C to 41.4 °C). Survival rates between high and medium doses were not significantly different (*p* = 0.075) from that between the high and low (*p* = 0.013) and medium compared to that of low (*p* = 0.017), which were significant. All the spleen samples in the infection groups collected post-euthanasia tested positive by ASFV qPCR, while in the high- and medium dose groups, ASFV positivity by PCR was detected much earlier in blood collected from 4 dpi. Postmortem examination of the organs showed hemorrhagic lymph nodes and fluid accumulation in the abdominal, thoracic, and pericardial cavities.

### RNA-Seq data quality check, mapping to pig host and ASFV pathogen reference genomes

Total RNA from 10 porcine splenic tissues was used for RNA-seq (3 each from medium, high, and low dose groups, and one animal from the control group). The total number of reads attained from the 10 locally-adapted pigs is summarized in Table 1. After QC, deduplication, and trimming off of multi-mapped reads, we retained an average of 45.3 M reads from the low dose pig samples, 12.89 M reads from the medium dose pig samples, and 9.66 M reads from the high dose pig samples (Table 1). There were no significant variations in the number of reads between the replicates.

**Table 1:** Mapping statistics to the pig and the ASF virus reference genomes

Study group	High dose	Medium dose	Low dose	Control
Number of biological replicates	3	3	3	1
Total trimmed reads	9,660,832	12,889,691	45,343,542	30,355,538
No of reads mapped to pig host	5,763,796	3,493,725	42,818,622	27,560,673
No of genes mapped to pig host (% of coding genes mapped)	14,544 (93.56%)	13,755 (88.5%)	15,543 (99.99%)	15,527 (99.88%)
No of reads mapped to ASFV pathogen	14,302	13,363	12,381	0
No of genes mapped to ASFV pathogen (% of coding genes mapped)	172 (98.86%)	174 (99.43%)	167 (95.43%)	0

**Fig. 2:**
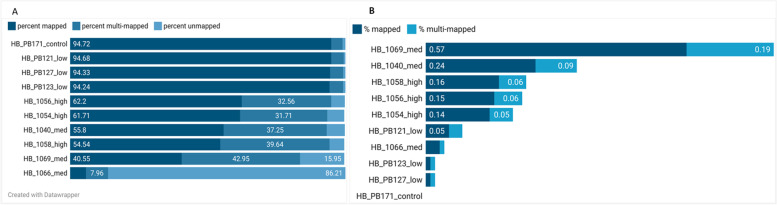
Mapping rates (**A**) to the pig and (**B**) to the ASF virus genomes. Created with Datawrapper on https://www.datawrapper.de/; accessed on 2^nd^ March 2022

Following the trimming of adaptors, deduplication, and removal of poor-quality reads, on average, 72.58% of the reads mapped to the pig genome (Fig. 2A). Pig HB_1066 from the medium dose group had very low mapping rates to the pig reference genome (5.75%) and was removed from the analysis. On average, of the trimmed data, 0.15% (min 0.01% and max 0.5%) of the reads mapped to the ASFV genome (Fig. 2B). The pigs challenged with the high- and medium doses had higher ASFV mapping rates, with pig HB_1069 from the medium dose group having the highest ASFV mapping rate of 0.5% (Fig. 2B).

### Differential gene expression in locally-adapted pig spleen tissues

**Fig. 3:**
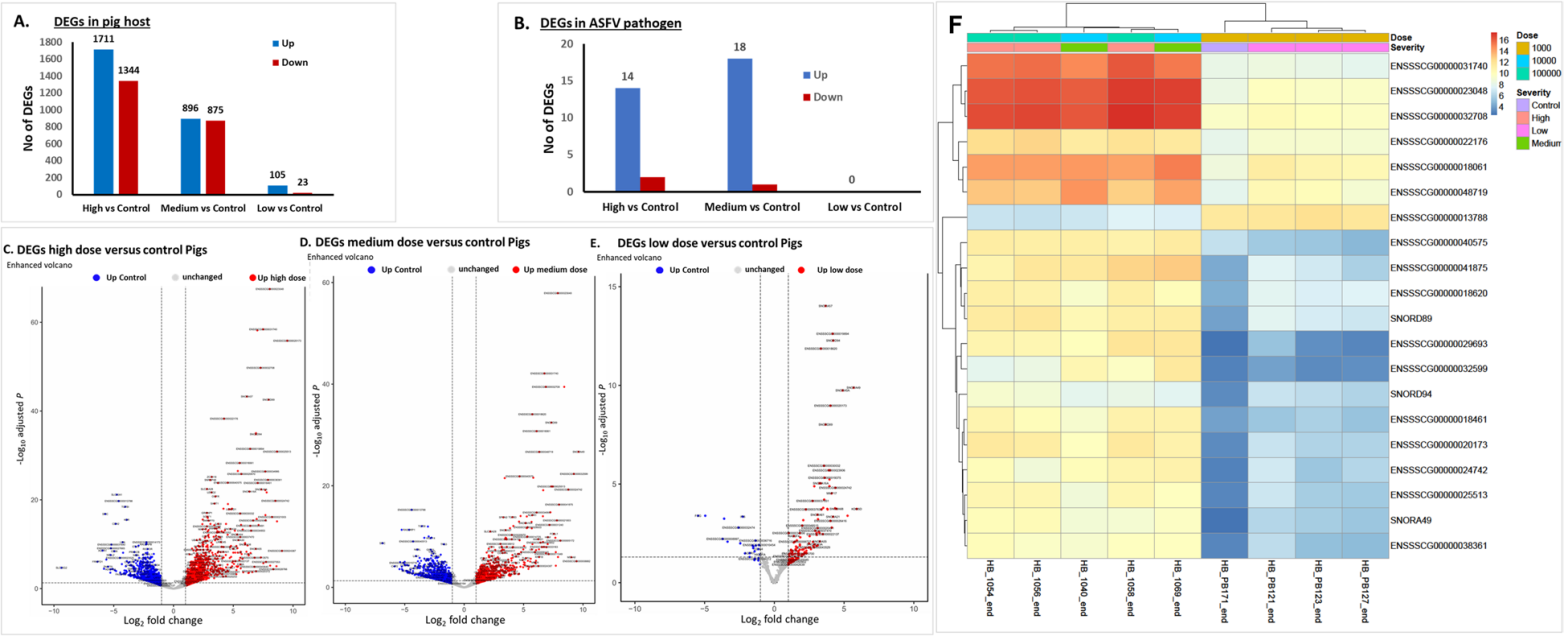
DEGs between three dose groups (**A**) in the pig host and (**B**) in the ASFV genes, and (**C**) Volcano plots for DEGs between high dose and control groups of pigs and (**D**) Volcano plots for DEGs between medium dose and control groups of pigs. (**E**) Volcano plots for DEGs between low dose and control groups of pigs. The dotted line across the volcano plots shows the cutoff for DEGs > 95% confidence (*p* < 0.05). (**F**) A heatmap of the DEGs

Compared to the control, we detected the expression of 15,543, 13,755 and 14,544 known pig genes in the low, medium, and high dose groups, respectively (Table 1 and Fig. 3). The complete list of pig genes detected by RNA-Seq is in Supplementary Table 2. We observed a wide variation in the gene count in transcripts per Million (TPM) of the expressed genes in the three infection groups, possibly due to differences in sample collection time points since these pigs reached humane end-point at different times (see survival curve, Fig. 1). We then selected genes with at least a 2-fold increase in gene expression relative to the control genes in at least two pigs as the top expressed pig genes. A summary of select differentially expressed genes (DEGs) is shown in Table 2. Of the 3055 DEGs detected in the high dose group, 1711 were upregulated while 1344 were downregulated (Fig. 3A), while the medium dose group had 896 DEGs upregulated and 875 downregulated. In the low dose group, 105 genes were upregulated while 23 were downregulated (Fig. 3A). In the high and medium dose groups, the top-upregulated pig host genes were associated with response to infection due to a highly pathogenic ASFV isolate. These DEGs could be divided into five groups, namely a) genes found on the macrophages, b) genes associated with natural killer cells, c) genes involved in ASFV infection, d) genes linked with the lymphocytes, e) other genes not linked to virus infection or immunity such as *DRAM2*, and *SOGA1*, are reported to be associated with autophagy. Other genes such as *TIMP1, LTF, CHP2, FOSL1,* and *FOXF1* play critical roles in viral pathogenesis [Table 2] [50, 51].

**Table 2:** List of select pig host genes detected in ASFV-infected pigs. Genes were selected based on function and mean fold change for three dose groups. The highest expressed fold change value between medium and high dose groups was recorded

Gene	log2 Fold Change	adjusted ***p***-value	Gene Product	Function	Reference
**Genes linked with monocyte macrophages**					
*CD163*	− 1.85	1.76E-02	CD163 antigen	A hemoglobin-specific receptor is found on the cell surface of macrophages involved in iron recycling and inflammatory response.	[52, 53]
*RGS16*	4.18	9.93E-19	Regulator of G Protein Signaling 16	Restricts pro-inflammatory response of monocytes	[54]
*HOX-1*	−2.13	2.06E-05	Heme oxygenase-1	Pro-oxidant and pro-inflammatory effects	[55, 56]
*S100A8*	7.03	3.01E-09	Calcyclin	Binds CD68	[57]
*CSF3R*	2.11	1.29E-02	Granulocyte colony-stimulating factor (G-CSF)	Involved in proliferation and differentiation of granulocytes	[58]
*TSP-1*	3.25	6.58E-04	Thrombospondin 1	Inhibits angiogenesis, regulates antitumor immunity and regulates extracellular proteases and growth factors.	[59]
**NK/T cell-associated genes**					
*IFIT2*	1.87	2.62E-04	Interferon-induced protein with tetratricopeptide repeats 2	IFN-induced antiviral protein which inhibits expression of viral messenger RNAs that lack 2′-O-methylation of the 5′ cap	[60]
*IFITM3*	3.05	2.80E-09	interferon induced transmembrane protein 3	Engages and shuttles the virus particles to lysosomes for clearance from the cells	[61]
**Genes associated with ASFV infection**					
*PPP1R15A*	2.59	7.49E-05	GADD34	Guides dephosphorylation of eIF2a by PP1	[62]
*SPIC*	− 6.87	2.02E-09	Spi-C Transcription Factor	Restrains inflammation and iron metabolism in macrophages	[63]
*NUPR1*	5.73	6.21E-15	Nuclear protein 1	Suppresses programmed cell death by apoptosis and programmed necrosis	[64]
**Genes associated with lymphocytes (B, T cells and NK cells)**					
*CD244*	−5.01	8.73E-03	CD244 molecule	Involved in NK cell stimulation and NK cell-mediated cytotoxicity	[65]
*CYFIP2*	− 3.05	1.06E-12	Cytoplasmic FMR1 interacting protein 2	Involved in T-cell adhesion and p53/TP53-dependent induction of apoptosis	[66]
*IL-6*	5.67	1.50E-26	Interleukin 6	Promotes virus survival and exacerbation of clinical disease	[67]
**Other genes**					
*DRAM2*	−2.39	9.44E-06	Damage-regulated autophagy modulator 2	Oncogenic regulator: promotes cell metastasis and proliferation in cancer cells	[50]
*SOGA1*	1.41	1.47E-03	Suppressor of glucose, autophagy associated 1	Implicated in autophagy	[51, 68]
*TIMP1*	5.27	2.17E-12	Tissue inhibitor matrix metallopeptidase inhibitor 1	Promotes tumorigenesis and metastasis of colon cancer and is a potential prognostic indicator for colon cancer	[69]
*LTF*	5.68	3.39E-08	Lactotransferrin	Sequestering iron and antimicrobial activity	[70]
*CHP2*	− 5.85	7.17E-05	Calcineurin like EF-hand protein 2	Regulates cell pH by controlling plasma membrane-type Na+/H+ exchange activity; involved in carcinoma progression	[71, 72]
*FOSL1*	7.55	2.94E-11	FOS like 1, AP-1 transcription factor subunit	Regulators of cell proliferation, differentiation, and transformation	[73]
*FOXF1*	2.37	3.23E-04	Forkhead box F1	FOXF1 transcription factor promotes lung regeneration	[74]
*PG-4*	7.11	2.84E-07	Protegrin-4	Antimicrobial peptide	[75]
*PGES-1*	4.22	1.02E-04	Prostaglandin E synthase	Plays a crucial role in inflammation by converting prostaglandin (PG) H2 to PGE2	[76]

Additionally, the Forkhead box (FOX) family of transcription factors (*FOXF1, FOXS1, FOXM1, FOXK1, FOXP4,* and *FOXC1*) play a role in cell differentiation and proliferation and are implicated in cancer and drug resistance [77]. These FOX transcription factors were all upregulated in the medium and high dose groups but not detected in the low dose group. The CSF3R is a type 1 cytokine receptor that binds the granulocyte colony-stimulating factor (G-CSF). G-CSF is a cytokine that is required for granulocyte proliferation and differentiation. *IFITM3* was significantly upregulated (over 3-fold), and it plays a critical role in immunity during viral infection in that it directly engages and shuttles incoming virus particles to lysosomes. We also detected the expression of *ATF4*, a transcription factor that upregulates genes involved in amino acid import, antioxidative stress response, and regulation of autophagy [78, 79].

Regarding genes associated with resistance/tolerance to ASF [25], the *RELA* proto-oncogene, NF-kB subunit, or transcription factor p65 (*RELA*) was detected at higher counts (2846 TPM) in the low dose group than the high and medium doses at 257 and 269 TPM, respectively (Supplementary Table 2). *RELA* was downregulated at − 1.94-fold (padj = 1.98E-04) and − 2.34-fold (padj = 4.71E-05) in the high- and medium dose groups but was not differentially expressed in the low dose group.

In terms of the genes associated with macrophage regulation [80], there was downregulation of the macrophage surface marker gene, *CD164* (decreased by 1.15-fold) in pigs that survived a low dose ASFV challenge. *HMOX1* codes for heme oxygenase-1, and its expression decreased by 1.89-fold and 1.46-fold in the high- and medium dose, respectively. The macrophage-associated genes were also upregulated in the medium and high dose groups, including *S100A4/A6/A8/A9/A13/A16* that play a role in Calcium-binding, innate immune response, and leukocyte migration associated with inflammatory response [81–83]. The angiopoietin-like protein (*ANGPTL1*) genes were highly differentially expressed at 1.61-fold and 5.02-fold in high- and medium dose groups. *CD244* is involved in the activation of NK cells leading to cell-mediated cytotoxicity [65] was downregulated in the high and medium dose groups by over 1.65 and 4.7-fold, respectively. Another differentially downregulated gene in medium and high dose groups was *CD36* at 3.89 and 4.91-fold change, respectively. CD36 is a scavenger receptor expressed in multiple cell types, mediates lipid uptake, immunological recognition, inflammation, molecular adhesion, and apoptosis, and binds thrombospondin-1 (*TSP-1*), resulting in attenuation of angiogenesis and induction of apoptosis/blocking the vascular endothelial growth factor receptor 2 (*EGFR2*) pathway in tumor microvascular endothelial cells [84]. We detected an upregulation of *TSP-1* in the high and medium dose groups by 1.92- and 3.25-fold change, respectively.

Antimicrobial peptides (AMPs) are molecules that possess a broad-spectrum activity against bacteria, fungi, protozoa, and viruses found in insects, amphibians, and mammals with antimicrobial, immunomodulatory and regulatory activity gut microbiota [85]. We detected AMPs of pharmaceutical value, such as protegrin-4 (*PG4*), a peptide isolated from porcine leucocytes [75]. *NPG4* was significantly upregulated in the medium dose (7.1-fold) and high dose (3.44-fold). The other upregulated genes were those involved in the host response to virus infection, such as *PPP1R15A* (protein phosphatase 1 regulatory subunit 15A), which codes for *GADD34* complex, a host protein involved in the dephosphorylation of P-eIF2α (Eukaryotic Initiation Factor 2) by an interferon-induced double-stranded RNA-activated protein kinase (*PKR*) in a prominent cellular antiviral pathway [62, 86]. Another essential gene expressed in macrophages is the *SpiC*, which we found to be the most downregulated gene in the high- and medium dose groups at 6.15-fold and 7.19-fold, respectively. *SpiC* plays a role in the downregulation of pro-inflammatory cytokines while promoting iron efflux by regulating ferroportin expression in activated macrophages [63].

CD163, a hemoglobin-specific receptor found on the cell surface of macrophages, is implicated in iron recycling and inflammatory response [52, 53], was downregulated. In contrast, *RSG16*, which restricts the pro-inflammatory response of monocytes [54], was significantly upregulated by over 4-fold in the medium and high dose groups. Expression of Prostaglandin E synthase (*PGES*) was upregulated in the medium and high dose groups. PGES plays a crucial role in inflammation by converting prostaglandin (PG) H2 to *PGE2* [87]. The matrix metalloproteinase (MMP) family catalyze proteolytic activities that result in the breakdown of the extracellular matrix [88]. MMPs thus play a key role in tumor invasion, neoangiogenesis, and metastasis [88]. The matrix metallopeptidase 17 (*MMP17*; or *MT4-MMP*) was highly upregulated (4.1-fold) in the low dose group compared to the high dose (1.84-fold). *MMP8* was upregulated in the medium and high dose groups (5.96- and 3.87-fold, respectively).

Nine cytokines showed significant differential expression from the high and medium dose groups (Table 3); eight of these were upregulated, and one was down-regulated. Interleukin-6 (*IL-6*), *VEGFA* and *IL27* were among the most upregulated genes by 5.67-, 3.81- and 2.2-fold, respectively. Seventeen cytokine receptors were differentially expressed, with *IL1RL1* being the most highly differentially expressed (5-fold), followed by *TNFRSF11A* (2.67-fold). *TNFRSF9* (TNF receptor superfamily member 9), also known as 4-1BBL or CD137, was most down-regulated by up to 3.11-fold in the medium dose. The interleukin cytokines IL6, IL27, and IL17B, were upregulated in the medium and high dose groups resulting in hemorrhagic fever by cytokine storm. There was an up-regulation of the proinflammatory cytokines involved in the apoptotic processes such as *TNF* (1.5-fold change), members of the *TWEAK* family such as *TNFSF12* (1.53-fold change), and *TNFSF13* (1.63-fold change). The IFN-ω has cross-species antiviral activities and was significantly downregulated in the high dose group (1.62-fold change). The immune-suppressive cytokine, *IL27*, was significantly upregulated in the high dose (2.2-fold) and medium dose groups (2.61-fold). We detected upregulation of the Interleukin-1 receptor (*IL-1R*) in the medium and high dose groups by over 5-fold in the high dose group and 4.01-fold in the medium dose group. We detected expression of interleukin-1 receptor-associated kinases (IRAKs) in the high dose group only where *IRAK1* and *IRAK3* were downregulated while expression of *IRAK4* was upregulated (Table 3).

**Table 3:** Differentially expressed cytokines and their receptors. TND = transcript not detected

Gene group	Gene	Description	Differential expression in High dose		Differential expression in medium dose		Differential expression in Low dose	
			Log2 Fold change	Adjusted *p*-value	Log2 Fold change	Adjusted *p*-value	Log2 Fold change	Adjusted *p*-value
**Cytokines**	*IL6*	Interleukin 6	**5.67**	1.50E-26	**4.01**	4.76E-11	TND	TND
	*IL17B*	Interleukin 17B	1.56	2.94E-02	TND	TND	TND	TND
	*IL16*	Interleukin 16	TND	TND	−2.08	4.17E-03	TND	TND
	*IL27*	Interleukin 27	**2.20**	4.28E-03	2.61	2.87E-03	TND	TND
	*IFN-ω*	Interferon omega 6	−1.62	3.22E-02	TND	TND	TND	TND
	*TNFSF12*	TNF superfamily member 12	1.53	1.01E-04	1.66	6.77E-05	TND	TND
	*TNFSF13*	TNF superfamily member 13	TND	TND	1.63	3.63E-03	TND	TND
	*HBEGF*	Heparin binding EGF like growth factor	1.83	4.07E-03	1.76	9.45E-03	TND	TND
	*VEGFA*	Vascular endothelial growth factor A	**3.81**	1.66E-13	2.83	1.12E-06	TND	TND
**Cytokine receptor families**	*IL1RL1*	Interleukin 1 receptor like 1	**5.00**	1.11E-09	**4.62**	2.58E-07	TND	TND
	*IL10RA*	Interleukin 10 receptor subunit alpha	−1.32	1.64E-03	−2.27	1.49E-06	TND	TND
	*IL15RA*	interleukin 15 receptor subunit alpha	1.54	4.86E-02	TND	TND	TND	TND
	*IL17RE*	Interleukin 17 receptor E like	1.64	2.14E-02	TND	TND	TND	TND
	*IL21R*	Interleukin 21 receptor	−2.61	1.44E-06	−2.78	3.62E-05	TND	TND
	*IL27RA*	Interleukin 27 receptor subunit alpha	TND	TND	−1.48	4.36E-03	TND	TND
	*IL31RA*	Interleukin 31 receptor A	−2.31	3.59E-03	**− 2.75**	5.42E-03	TND	TND
	*TNFRSF11A*	TNF receptor superfamily member 11a	**−2.67**	5.45E-04	−2.05	1.24E-02	TND	TND
	*TRAF7*	TNF receptor associated factor 7	1.59	4.75E-05	TND	TND	TND	TND
	*TNFRSF13B*	TNF receptor superfamily member 13B	2.34	8.13E-07	TND	TND	TND	TND
	*TNFRSF18*	TNF receptor superfamily member 18	1.99	3.68E-03	TND	TND	TND	TND
	*TNFRSF9*	TNF receptor superfamily member 9	−1.70	3.90E-02	**−3.16**	9.69E-03	TND	TND
	*TNFRSF21*	TNF receptor superfamily member 21	−1.92	1.19E-03	−1.42	2.09E-02	TND	TND
	*TNFRSF13C*	TNF receptor superfamily member 13C	TND	TND	−2.33	7.88E-03	TND	TND
	*IRAK1*	Interleukin 1 receptor associated kinase 1	1.73	2.26E-06	TND	TND	TND	TND
	*IRAK3*	Interleukin 1 receptor associated kinase 3	−0.87	3.75E-02	TND	TND	TND	TND
	*IRAK4*	Interleukin 1 receptor associated kinase 4	−1.22	1.67E-02	TND	TND	TND	TND

In the spleen, chemokines are essential in modulating adaptive immune response by promoting the initial priming of lymphocytes and guiding their differentiation and phenotype. There were nine differentially expressed chemokines (Table 4). The C-C ligand 2 (CCL2) and C-X-C motif chemokine ligand 2 (*CXCL2*), *CXCL10, CCL23, CCL4,* and *CXCL8* were significantly upregulated in the medium dose group, while in the high dose group, *CCL2, CCL4, CXCL2* and *CXCL10* were significantly upregulated. *CCL21, CCL26* and *CXCL13* were significantly downregulated in both the high and medium dose groups (Table 4). *CCL4* and *CXCL10*, the chemo-attractants for immune response, were upregulated in the medium and high dose groups, while *CXCL10* was downregulated in the low dose group. *CCL2* was the most upregulated chemokine (4.03- and 4.11-fold), while *CCL26* and *CXCL13* were the most downregulated in the high (5.28- and 2.5-fold) and *CCL21* and *CCL26* in the medium dose groups (4.30- and 4.17-fold). *CXCL2* and *CXCL8* are involved in recruiting neutrophils. The ELR+ (glutamic acid – leucine – arginine) CXC chemokines *CXCL2* were significantly upregulated in the medium (2.68-fold) and high (3.44-fold) dose groups. *CXCR2* signaling is essential for neutrophil release from the bone marrow into the blood.

**Table 4:** Differentially expressed chemokines and their chemotactic activities. TND = transcript not detected

Chemokine	Key immune function	Differential expression in High dose		Differential expression in medium dose		Differential expression in Low dose		Chemotactic activity
		log2 Fold Change	padj	log2 Fold Change	padj	log2 Fold Change	padj	
*CCL2*	Inflammatory monocyte trafficking	4.03	3.76E-10	4.11	1.12E-08	TND	TND	Classical monocyte, Natural killer cells
*CCL4*	Macrophage and natural killer cell migration; T cell–dendritic cell interactions	1.69	2.30E-02	TND	TND	TND	TND	Nonclassical-Monocyte, Natural killer cells
*CCL21*	T cell and dendritic cell homing to lymph node	TND	TND	−4.30	6.14E-04			Neutrophils, CD8+ T cells, dendritic cells
*CCL26*	Eosinophil and basophil migration	−5.28	2.51E-04	−4.17	3.45E-03	TND	TND	Th2 type T lymphocytes
*CXCL2*	Neutrophil trafficking	3.44	9.55E-06	2.68	1.70E-03	TND	TND	Neutrophils, CD8+ T cells, monocytes/macrophage, natural killer cells
*CXCL8*	Neutrophil trafficking	TND	TND	2.42	1.22E-02	TND	TND	Neutrophils, CD8+ effector T cells, monocytes/macrophage, natural killer cells
*CXCL10*	Th1 response; Th1, CD8, NK trafficking	1.62	8.29E-03	2.85	4.93E-05	−2.56	1.61E-03	CD8+ T cells, T helper cells (Th1), natural killer cells
*CXCL13*	B cell and follicular helper (Tfh) cell positioning in lymph node	−2.52	2.35E-03	−1.67	4.13E-02	TND	TND	B cells

Antigen processing and presenting cells were downregulated in the medium and high dose groups. The differentially expressed genes involved in MHC antigen processing and presentation are listed in Table 5. The expression of *SLA-DMB, SLA-DQA, SLA-DRA, SLA-DRB,* and *SLA-DOB* were down-regulated in the medium and high dose groups. The cathepsin S was downregulated among the medium and high dose groups, compromising the antigen presentation by MHC class II molecules. Cathepsin S (*CTSS*) gene is involved in processing antigens before loading to MHC class II and was significantly downregulated (2.10-fold change). The proteasome activators (*PSMC, PSMD, PSME, PSMF*) were upregulated in the medium and low dose groups.

**Table 5:** Differentially expressed genes involved in MHC antigen processing and presentation. TND = transcript not detected

Gene	Description	Differential expression in High dose		Differential expression in medium dose		Differential expression in Low dose	
		log2 Fold Change	padj	log2 Fold Change	padj	log2 Fold Change	Padj
*SLA-DQB1*	SLA-DQ beta1 domain	−1.36	4.51E-03	−1.64	9.01E-04	TND	TND
*SLA-DQA*	MHC class II histocompatibility antigen SLA-DQA	−1.85	8.57E-04	−1.19	3.97E-02	TND	TND
*SLA-DRA*	MHC class II DR-alpha	−1.62	5.34E-03	TND	TND	TND	TND
*SLA-DRB1*	MHC class II histocompatibility antigen SLA-DRB1	TND	TND	−1.20	4.72E-02	TND	TND
*SLA-DOB*	MHC class II, DO beta	TND	TND	−2.37	2.33E-03	TND	TND
*SLA-DMB*	MHC class II, DM beta	−1.67	1.84E-03	−1.27	2.28E-02	TND	TND
*SEL1L3*	SEL1L family member 3	1.59	4.11E-02	TND	TND	TND	TND
*SEL1L*	SEL1L adaptor subunit of ERAD E3 ubiquitin ligase	−2.13	1.43E-05	−1.47	5.07E-03	TND	TND
*PSME4*	Proteasome activator subunit 4	−0.96	7.83E-03	TND	TND	TND	TND
*PSMD4*	Proteasome 26S subunit, non-ATPase 4	TND	TND	1.29	3.23E-03	TND	TND
*PSMD3*	Proteasome 26S subunit, non-ATPase 3	1.32	4.70E-03	TND	TND	TND	TND
*PSMC5*	Proteasome 26S subunit, ATPase 5	TND	TND	0.89	2.78E-02	TND	TND
*PSMC3*	Proteasome 26S subunit, ATPase 3	TND	TND	1.16	3.35E-02	TND	TND
*PSMC1*	Proteasome 26S subunit, ATPase 1	TND	TND	1.10	2.25E-02	TND	TND
*PMSF1*	Proteasome inhibitor subunit 1	0.98	3.08E-02	1.10	1.61E-02	TND	TND
*CTSS*	Cathepsin S	−2.10	5.96E-06	−1.88	2.60E-04	TND	TND
*ADRM1*	ADRM1 26S proteasome ubiquitin receptor	1.20	1.60E-02	TND	TND	TND	TND

In total, we detected 10 autophagy-related genes in the medium and high dose study groups and only one was detected in the low dose group. Seven of these were downregulated, namely *ATG4C, DRAM2, DCT, EPG5, APAF1, NBR1* and *FUNDC1* (Table 6). Autophagy-associated cell death is inhibited by the nuclear protein 1 (NUPR1), a transcriptional regulator gene that was significantly upregulated in the high and medium dose groups by over 5-fold (Tables 2 and 6). In the high and medium dose groups, we also detected the upregulation of pro-apoptosis and an autophagy inducer gene, BCL2 interacting protein 3 (BNIP3). Twelve [12] other autophagy and cell death regulating genes were detected, of which 10 were upregulated, including *FAIM2, GAS2L1, GAS2L2, GAS8, MAD1L1, MAD1L2, GADD45G, GADD45B, GAS7,* and *NUPR1*. Five of these were significantly upregulated (*FAIM2, GAS2L2, GAS8, MAD1L1, and GADD45G*). *GAS2L3* (Growth arrest-specific 2 like 3) and *APAF1* were downregulated, with *GAS2L3* being significantly downregulated (Table 6).

**Table 6:** Differentially expressed genes associated with autophagy. TND = transcript not detected

	Gene	Description	Differential expression in High dose		Differential expression in medium dose		Differential expression in Low dose	
			log2 Fold Change	padj	log2 Fold Change	padj	log2 Fold Change	Padj
**Autophagy-related Genes**	*ATG4C*	Autophagy related 4C cysteine peptidase	−1.39	1.64E-02	TND	TND	TND	TND
	*ATG4D*	Autophagy related 4D cysteine peptidase	1.10	4.64E-02	TND	TND	TND	TND
	*BNIP3*	BCL2 interacting protein 3	3.09	2.93E-09	2.53	1.72E-05	TND	TND
	*DCT*	Dopachrome tautomerase	TND	TND	−1.94	4.41E-02	TND	TND
	*DRAM2*	DNA damage regulated autophagy modulator 2	**−2.39**	9.44E-06	−1.58	5.40E-03	TND	TND
	*EPG5*	Ectopic P-granules autophagy protein 5 homolog	TND	TND	−1.38	2.98E-02	TND	TND
	*FXR2*	FMR1 autosomal homolog 2	TND	TND	1.01	1.50E-02	TND	TND
	*NBR1*	NBR1 autophagy cargo receptor	−1.25	1.41E-03	TND	TND	TND	TND
	*SOGA1*	Suppressor of glucose, autophagy associated 1	1.41	1.47E-03	TND	TND	TND	TND
	*SSNA1*	SS nuclear autoantigen 1	1.23	2.04E-02	1.13	3.81E-02	TND	TND
	*FUNDC1*	FUN14 domain containing 1	TND	TND	TND	TND	−0.96	3.69E-02
**Autophagy and cell death regulating genes**	*GAS2L2*	Growth arrest-specific 2 like 2	**4.93**	1.26E-06	TND	TND	TND	TND
	*GAS2L1*	Growth arrest specific 2 like 1	1.31	4.10E-02	1.39	2.98E-02	TND	TND
	*GAS2L3*	Growth arrest specific 2 like 3	TND	TND	**−2.32**	2.45E-02	TND	TND
	*GAS7*	Growth arrest specific 7	1.86	5.02E-05	TND	TND	TND	TND
	*GAS8*	Growth arrest specific 8	**3.74**	2.60E-15	1.52	4.38E-03	TND	TND
	*MAD1L1*	Mitotic arrest deficient 1 like 1	**3.30**	2.53E-08	TND	TND	TND	TND
	*MAD1L2*	Mitotic arrest deficient 1 like 2	1.64	9.99E-03	TND	TND	TND	TND
	*GADD45G*	Growth arrest and DNA damage inducible gamma	2.25	1.39E-02	2.11	3.35E-02	TND	TND
	*GADD45B*	Growth arrest and DNA damage inducible beta	1.73	1.33E-02	2.00	8.46E-03	TND	TND
	*FAIM2*	Fas apoptotic inhibitory molecule 2	TND	TND	3.96	2.87E-03	TND	TND
	*NUPR1*	Nuclear protein 1, transcriptional regulator	**5.13**	8.83E-14	**5.73**	6.21E-15	TND	TND
	*APAF1*	Apoptotic peptidase activating factor 1	**−1.49**	6.29E-03	TND	TND	TND	TND

In total, we detected 57 signal transduction and transcription genes required for macrophage activation in the medium and high dose groups (Supplementary Table 3). Twenty-seven of them were upregulated, with 9 being significantly upregulated (> 2-fold change). Of the 30 downregulated signal transduction and transcription genes, nine (*USP34, USP44, USP45, USP37, MAP2K6, MAP3K2, IRF4, MEF2C,* and *MEF2B*) were significantly downregulated (> 2-fold change). Two CCAAT enhancer-binding proteins (or CEBPs) were upregulated in the medium and high dose groups, namely *CEBPD* and *CEBPB*. TAB3 expression was detected, and five other key immune transcription factors (*FOSB, FOSL1, FOSL2, IRF7, JUNB, IRF4,* and *IRF8*) were all upregulated except for IRF4 and IRF8, which were downregulated in the high and medium dose groups (Supplementary Table 3). Also detected was the expression of ten ubiquitin-specific peptidases (USP) and three suppressors of cytokine signaling (*SOCS*), namely U*SP1, USP14, USP20, USP24, USP25, USP33, USP34, USP35, USP37, USP4, USP44, USP45, USP47, USP48, USP7, USP9X, SOCS3, SOCS4* and *SOCS7*. The expression of *SOCS3, SOCS7, USP14, USP20,* and *USP35* was upregulated, while the rest were downregulated in the high dose group.

In total, we detected the expression of 20 mitogen-activated protein kinase (MAPK), namely *MAP2K3, MAP2K4, MAP2K6, MAP3K1, MAP3K10, MAP3K14, MAP3K2, MAP3K5, MAP3K6, MAP3K9, MAP4K2, MAP4K4, MAPK1, MAPK6, MAPK7, MAPK8, MAPK8IP3, MAPK9, MAPKAP1,* and *MAPKAPK2*. Of these, *MAPK7, MAP3K6, MAP3K10, MAP4K4, MAP2K3, MAP3K14, MAP3K9, MAPK8IP3, MAP3K5* and *MAPKAPK2* were upregulated, while *MAP3K1, MAP2K4, MAPK1, MAPK6, MAPK8, MAPK9, MAP3K2, MAP2K6, MAP4K2,* and *MAPKAP1* were downregulated. MAPK cascades are signaling pathways that regulate cellular processes, such as proliferation, differentiation, apoptosis and stress responses crucial for cancer development and progression [89].

Small nucleolar RNAs (SnoRNAs) exhibit oncogenic and tumor-suppressive actions vital in lung cancer tumorigenesis and progression by participating in the invasion of growth suppressors and cell death, activation of invasion and metastasis angiogenesis, and continued proliferative signaling [90]. In this study, we detected differential expression of several small SnoRNAs in all three study groups at 10.32- and − 2.54-fold across the groups. 42, 33, and 30 SnoRNAs were differentially expressed in the high, medium and low dose groups, respectively. The downregulated SnoRNAs were 2 in the high dose group and 3 in the low dose group. The small nucleolar RNA, C/D box 45A, was downregulated in both the medium and high dose groups.

### ASFV gene expression in infected spleen tissues

In total, we detected the expression of 172 (high dose), 174 (in medium dose), and 167 (low dose) known ASFV genes and multigene families (MGFs) in the spleen samples analyzed from the locally-adapted pigs (Table 1). The complete list of ASFV genes detected is found in Supplementary Table 4. A total of 44 known MGFs were detected in reference to the Ken06.Bus ASFV genome Supplementary Table 5. We observed a wide variation in the gene count in Transcripts per Million (TPM) of the expressed genes in the different pigs studied, probably due to variations in the time the pigs were euthanized and variations in the number of infected macrophages at the time of euthanasia. In ASFV, higher gene counts of MGFs were detected in pigs from the high- and medium dose groups (Supplementary Table 5). The uncharacterized protein (*C84L*), viral DNA polymerase (*G1211R*), polyprotein pp220 (CP2475L), and a hypothetical protein (*ASFV_G_ACD_00190*) were the top four genes in the low dose group with 204, 123, 109, and 101 TPM being detected. In the medium dose group, MGF_100-1 L, Uncharacterized protein (*E184L*), and the structural protein p72 (*B646L*) were expressed in 2097, 1533, and 1271 TPM, respectively. The top 10 genes with the highest counts in TPM were *MGF 100-1 L, E184L, B646L, B385R, I196L, MGF 360-4 L, NP1450L, MGF 360-1La, I215L,* and *K145R* (Table 7). The gene *L83L* interacts with the host IL-1R and was detected in high dose (13 TPM), medium dose (45 TPM) and low dose (6 TPM) [Supplementary Table 4]. *E184L, B646L,* and *MGF_100-1 L* were expressed at 1004, 1001, and 862 TPM in the high dose group. The top three genes in the medium and high dose were the same, with differences in the number of TPM.

**Table 7:** Top 20 ASFV genes by counts in TPM

Gene	Gene counts (TPM) High dose	Gene counts (TPM) Medium dose	Gene counts (TPM) Low dose
MGF 100-1 L	862	2097	20
E184L	1004	1533	18
B646L	1001	1271	18
B385R	436	1185	18
I196L	516	1155	10
MGF 360-4 L	376	989	21
NP1450L	648	917	53
MGF 360-1La_CDS	297	864	5
I215L	369	781	73
K145R	434	735	43
E301R	313	719	10
C962R	395	701	47
MGF 360-21R	229	696	21
MGF 360-6 L	226	689	40
A151R	265	653	4
M448R	260	651	21
B354L	305	631	81
CP312R	341	623	15
H339R	259	588	16
MGF 360-15R	286	563	34

**Fig. 4:**
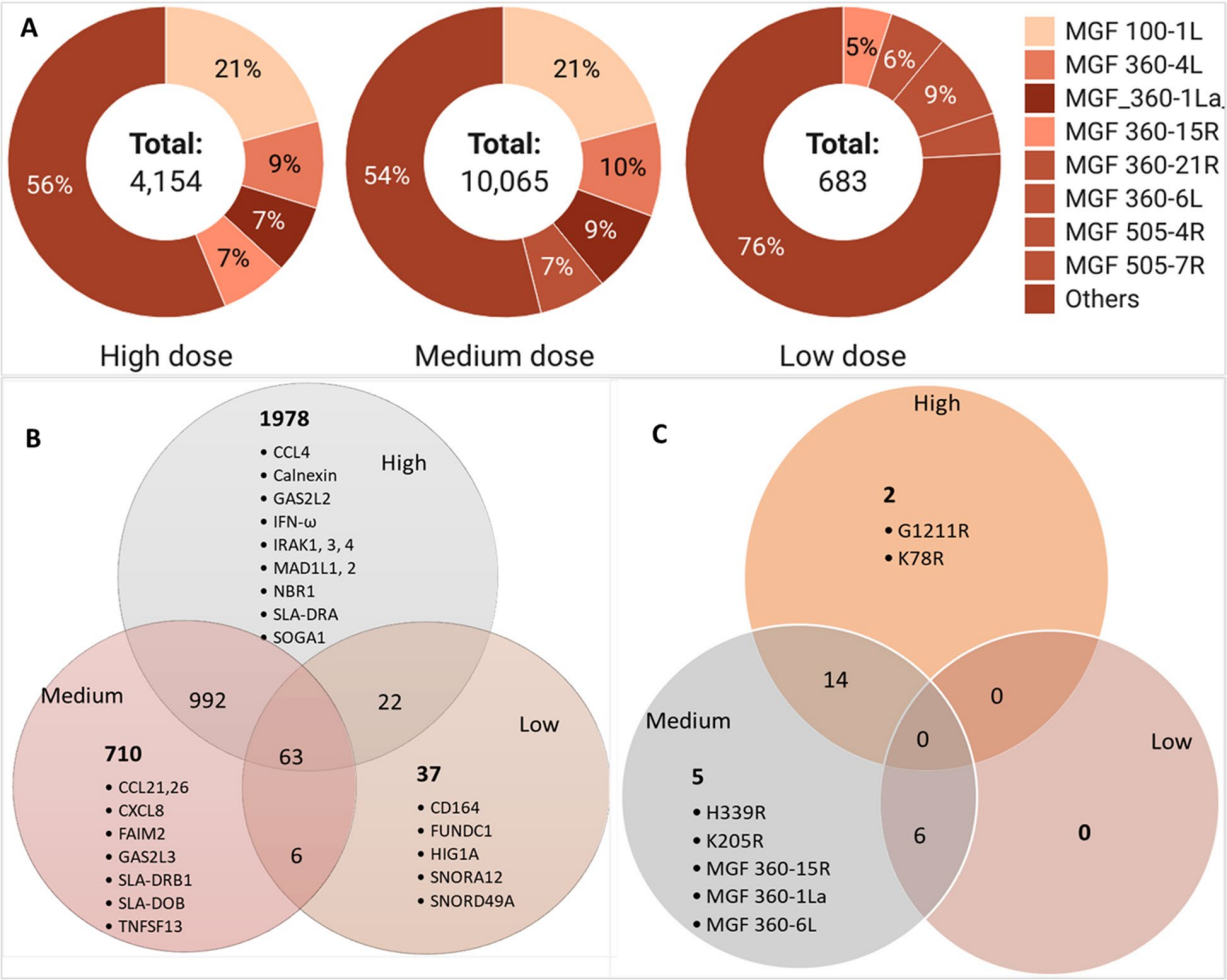
**A** Gene counts (in TPM) of ASFV Multigene families (MGFs), in high, medium and low dose groups, created with *Datawrapper* on https://www.datawrapper.de/; accessed on 2nd March 2022, (**B**) Venn diagram of shared DEGs in the pig host and (**C**) Venn diagram of shared DEGs in the ASFV pathogen

MGFs demonstrate divergence in sequence, indicating they have evolved over long periods and thus offer a selective advantage to the virus [91]. This study detected several MGFs and the top 10 MGFs detected were *MGF_100-1 L, MGF_360-4 L, MGF_360-1La, MGF_360-6 L, MGF_360-21R, MGF_360-15R, MGF_100-3 L, MGF_505-4R, MGF_505-1R* and *MGF_110-7 L* (Supplementary Table 5). These MGFs were detected at very low gene counts (in TPM) in the low dose group (Supplementary Table 5 and Fig. 4) compared to the medium and high dose groups (Fig. 4A). The highest gene counts in the low dose group were for *MGF 505-4R* (62 TPM), *MGF 360-6 L* (40 TPM), and *MGF 360-15R* (34 TPM). The highest gene counts in the high and medium dose groups were for *MGF 100-1 L* (862, 2097 TPM), *MGF 360-4 L* (376, 2097 TPM), and *MGF_360-1La* (297, 864 TPM). The MGFs (MGF 360-10 L, 11 L, 12 L, 13 L, 14 L, and MGF505-1R, 2R, 3R) have been associated with virulent ASFV isolates [91] and were detected in all the three study groups affirming the virulence of the ken12/busia.1 ASFV isolate (Supplementary Table 5).

We then selected genes with a 2-fold increase in gene expression relative to the control genes in the control animal (Table 8). The highest mean expression levels were detected in *E184L*, *MGF 100-1 L* and *NP1450L*, all in the high and medium dose groups. *MGF 100-1 L* was recently shown to be highly expressed in ASF surviving pigs [92]. We detected ASFV structural, non-structural, and host regulatory genes and genes of unknown function (Supplementary Table 3). We detected *MGF 360–15R* (*A276R*) expression that is vital in blocking early innate immune responses by inhibiting the induction of IFN-β [93]. Also detected were ASFV structural genes such as *CP204L*, which encodes p30, an immunodominant phosphoprotein of the virion and a preferred target for ASFV serological detection of infection. Another structural protein also detected is P72 (B646L), a major capsid protein involved in virus entry and a major molecular marker for distinguishing and genotyping ASFV isolates. Additionally, the structural protein P54 (*E183L*) was also differentially expressed. P54 binds the LC8 chain of dynein, and is involved in virus entry, and is also required to recruit envelope precursors. We detected the other structural genes, such as *KP177R* (p22), *A04R* (histone-like), *A151R* (pA151R), and *EP402R*, which are similar to the pig host CD2 protein that is required to bind the host red blood cells to infected cells and extracellular particles resulting in haemadsorption to infected cells [16]. *EP402R* had fewer gene counts in the low dose group (36 TPM) compared to the medium (234 TPM) and high dose [94] groups. The expression of ASFV A238L was repressed in the high and medium dose groups compared to the low dose groups with gene counts of 18, 2, and 0 TPM, respectively. Another ASFV gene critical in host-pathogen interaction is *A224L*, an apoptosis inhibitor, which was detected at high amounts in the high and medium dose groups (172 and 303 TPM, respectively) compared to the low dose groups (13 TPM); however, it was not differentially expressed (Supplementary Table 4).

**Table 8:** Differentially expressed ASFV genes in high and medium dose groups. TND = transcript not detected

ORF (Gene)	Differential expression in High dose		Differential expression in medium dose		Differential expression in Low dose		Description/Function	Reference
	log2 Fold Change	Padj	log2 Fold Change	padj	log2 Fold Change	padj		
A151R	2.68	2.74E-02	2.99	1.60E-02	TND	TND	Inhibits Absent in Melanoma 2 (AIM2) inflammasome activation.	[95]
A240L (TK)	2.10	4.80E-02	2.38	4.63E-02	TND	TND	Thymidylate kinase Involved viral in DNA synthesis	[96]
B646L (p72)	2.34	4.80E-02	2.25	4.76E-02	TND	TND	Encodes the variable major capsid protein, p72, an immunogenic protein in natural infections. Involved in virion assembly and entry. Late transcription gene.	[97, 98]
C84L	−2.74	7.99E-03	−2.82	8.18E-03	TND	TND	Uncharacterised protein	[99]
CP204L (p30)	2.23	4.80E-02	TND	TND	TND	TND	Plays a role in virus cell tropism, and is essential for effective virus entry and replication in macrophages.	[10]
E184L (j12L)	2.13	4.80E-02	2.25	4.76E-02	TND	TND	Uncharacterised protein	
EP402R (CD2v)	2.08	4.81E-02	2.28	4.76E-02	TND	TND	Similar to host CD2 protein, needed for binding red blood cells to infected cells and extracellular virus particles; responsible for heamadsorption in infected cells; glycoprotein inserted into external virus envelope	[100]
I177L (k14L)	2.13	4.80E-02	2.61	3.54E-02	TND	TND	Uncharacterised protein	[100]
I196L (k15L)	2.15	4.80E-02	2.37	4.72E-02	TND	TND	Uncharacterised protein	[100]
I215L (k13L)	TND	TND	1.51	4.76E-02	TND	TND	Ubiquitin-conjugating enzyme	[100]
I73R (k10R)	2.24	4.80E-02	2.59	3.54E-02	TND	TND	Uncharacterised protein	[100]
K78R (p10)	2.05	4.97E-02	TND	TND	TND	TND	DNA-binding protein p10 involved in morphogenesis	[100]
MGF 100-2 L	2.82	2.45E-02	3.22	1.18E-02	TND	TND	Modulate host cell functions	[91]
MGF 100-3 L	2.79	2.45E-02	3.15	1.18E-02	TND	TND		
MGF 360-15R	2.55	3.57E-02	2.78	2.79E-02	TND	TND		
MGF 360-22R	2.21	4.80E-02	2.48	3.85E-02	TND	TND		
MGF 360-4 L	2.08	4.81E-02	2.50	3.85E-02	TND	TND		
NP1450L (g2L)	2.26	4.80E-02	2.28	4.76E-02	TND	TND	RNA polymerase subunit 1 is involved in transcription	[98]

Additional genes differentially expressed were those that code for immunodominant ASFV proteins, namely *E184L, CP312R, K205R,* and *K145R* [45]. Other genes detected in higher counts in the medium and high dose groups are associated with late ASFV Infection, such as *NP1450L* and *EP1242L* [101]. Another late ASFV infection gene detected was *S273R*, which codes for SUMO-1-specific proteases, that cleave the viral polyproteins pp62 and pp220 [102]. *S273R* was detected at very low gene counts (< 20 TPM) in all the study pigs (Supplementary Table 4).

### Overlaps in differential gene expression in the spleen

Of the 4954 DEGs, overlaps in differential gene expression between the low-, medium-, and high dose groups were detected. In the pig host, 992 (20%) genes were shared between medium and high dose groups, and 22 (< 1%) genes were shared between high and low dose groups (Fig. 4B and Supplementary Table 7). A total of 63 (1%) DEGs were shared between the three study groups. Some of the uniquely expressed genes in the pig host in the high dose group were *CCL4, Calnexin, GAS2L2, IFN-ω, IRAK1, IRAK3, IRAK4, MAD1L1, MAD1L2, NBR1, SLA-DRA, ATG4C, ATGD* and *SOGA1* (Fig. 4A). In the medium dose group*, CCL21, CCL26, CXCL8, FAIM2, GAS2L3, SLA-DRB1, SLA-DOB,* and *TNFSF13* were among the uniquely expressed genes. While in the low dose group, the expression of *CD164, FUNDC1, HIG1A, SNORA12,* and *SNORD49A* were unique to this study group. In the ASFV pathogen, 15 DEGs were shared between the medium and high dose groups (Fig. 4C). Two genes were unique to the high dose [CP204L (p32%2C p30) and K78R)], while the medium dose group had one unique ASFV DEG [I215L (k13L)].

### Mapping pig genes to KEGG pathways

The pig and human gene atlases were used to link host gene expression with cells and tissues. The pathways containing the most significant number of genes represented were functionally characterized using the DAVID gene enrichment tool to report KEGG pathways. The medium dose group had the highest number of significantly enriched pathways (*n* = 11; Table 9). The pathways with the highest gene counts were linked to immune response functions primarily associated with the host immune response to viral infection. In the medium dose group, pathway hsa04060, cytokine-cytokine receptor interaction, had the highest number of highly upregulated genes (*n* = 24), and the upregulated genes were ssc04657 (IL-17 signaling pathway), ssc04061, (viral protein interaction with cytokine and cytokine receptor) and ssc04657 (IL-17 signaling pathway). The above three pathways contained highly upregulated genes that represented the class 1 helical cytokines (IL6, IL27, CSF3 and IL15RA), IL17-like cytokines (*IL17B* and *ILA7RA*), the CC- and CXC-subfamily of chemokines (*CCL2, CXCL2, CXCL10, CCL23, CCL4, CXCL8,* and *CXCL13*) and TNF family (*TWEAK* and *TNFR1*).

**Table 9:** The KEGG significantly enriched pathways in the three infective doses (high, medium, and low)

	ID	Description	p.adjust	qvalue	Count
High vs. control	ssc04610	Complement and coagulation cascades	8.42E-03	8.17E-03	12
	ssc05323	Rheumatoid arthritis	2.89E-02	2.80E-02	12
	ssc04657	IL-17 signaling pathway	3.37E-02	3.27E-02	12
Medium vs. control	ssc04060	Cytokine-cytokine receptor interaction	4.12E-04	3.81E-04	24
	ssc05323	Rheumatoid arthritis	4.12E-04	3.81E-04	14
	ssc04061	Viral protein interaction with cytokine and cytokine receptor	4.12E-04	3.81E-04	13
	ssc05340	Primary immunodeficiency	1.37E-03	1.26E-03	9
	ssc04640	Hematopoietic cell lineage	1.40E-03	1.29E-03	13
	ssc05144	Malaria	1.68E-03	1.56E-03	10
	ssc04080	Neuroactive ligand-receptor interaction	1.80E-02	1.66E-02	17
	ssc04672	Intestinal immune network for IgA production	1.80E-02	1.66E-02	8
	ssc05142	Chagas disease	1.80E-02	1.66E-02	13
	ssc04657	IL-17 signaling pathway	1.80E-02	1.66E-02	11
	ssc04514	Cell adhesion molecules	2.28E-02	2.11E-02	14
Low vs. control	ssc04610	Complement and coagulation cascades	5.04E-03	4.60E-03	3
	ssc05171	Coronavirus disease - COVID-19	7.38E-03	6.73E-03	4
	ssc04611	Platelet activation	1.38E-02	1.26E-02	3
	ssc04613	Neutrophil extracellular trap formation	1.38E-02	1.26E-02	3

In the surviving low dose group, four pathways were significantly enriched in which all represented genes were significantly downregulated: ssc04610 (complement and coagulation cascades), ssc05171 (Coronavirus disease, COVID-19), ssc04611 (platelet activation), ssc04613 (neutrophil extracellular trap formation). The following genes were significantly downregulated in the complement and coagulation cascade, platelet activation and neutrophil extracellular trap formation pathway: FGG (fibrinogen gamma chain) plays a crucial role in pathophysiologic processes, such as inflammation and thrombosis [102] was highly downregulated by 5.47-fold. Other genes in the complement and coagulation cascades were FGA (fibrinogen alpha chain) downregulated at 4.96-fold and fibrinogen beta chain suppressed by over 3.7-fold. In the COVID-19 pathway, the CXCL10 chemokines were highly downregulated in the low dose group by over 2.56-fold.

The Hippo signaling pathway that controls animal organ size through cell proliferation and apoptosis regulation, including cell proliferation, apoptosis, and various stress responses [103], was significantly enriched in the medium and high dose groups (Supplementary Figs. 1 and 2). The tumor promotor Hippocalcin 1 (HPCAL1) was differentially expressed by 1.54 and 1.93-fold change in the medium and high dose groups, respectively, resulting in the observed organomegaly [103] among pigs in the high and medium dose groups following a postmortem. The hypoxia-inducible pathways were also significantly enriched, such as the HIF-1 signaling pathway that mediates adaptive responses to oxygen deprivation [104], typical in ASF infection.

In the low dose group, the enriched pathways were in response to viral carcinogenesis, thermogenesis, antigen processing and presentation, protein synthesis and metabolic activities (Supplementary Figs. 3 A, B and C). All the genes in the KEGG enriched terms in the low dose group were downregulated. HIG1 hypoxia inducible domain family member 1A (*HIGD1A*) gene was downregulated in the surviving low dose group only by 1.02-fold.

### Functional annotation

When comparing the enriched GO terms, we detected 139 terms, of which 115 represented the biological processes (CC), 3 were cellular components (CC), and 21 molecular functions (MF) [Supplementary Table 6]. The GO terms: response to external stimulus (GO:0009605), extracellular region (GO:0005576), and cellular response to an organic substance (GO:0071310) had the highest gene counts in the three infective groups studied. The GO terms response to external stimulus (GO:0009605), extracellular region (GO:0005576), cellular response to an organic substance (GO:0071310), defense response (GO:0006952), the biological process involved in interspecies interaction between organisms (GO:0044419), response to external biotic stimulus (GO:0043207), response to other organisms (GO:0051707), and inflammatory response (GO:0006954) were detected in the high and medium dose groups only. In the low dose, there were less than 5 gene counts represented in these GO terms: extracellular region (GO:0005576), signaling receptor binding (GO:0005102), extracellular space (GO:0005615), cytokine receptor binding (GO:0005126), heme-binding (GO:0020037), hydrogen peroxide metabolic process (GO:0042743), tetrapyrrole binding (GO:0046906). The last three terms were detected only in the low dose group.

**Fig. 5:**
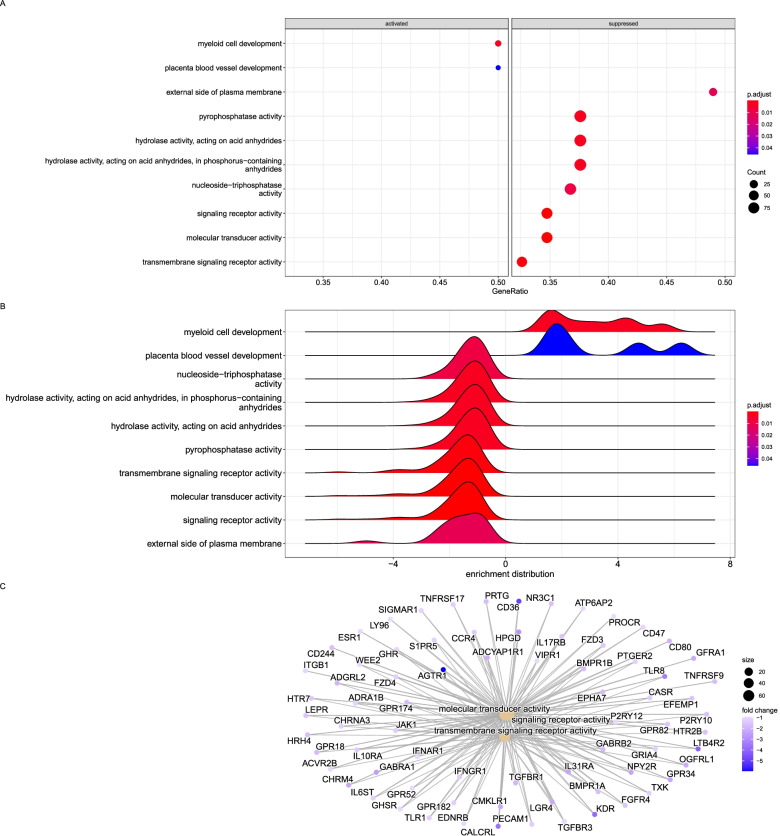
Top enriched KEGG pathways and GO annotations of the DEGs identified from the high dose group. **A**. Overrepresented gene ratios of significantly expressed genes activated or suppressed in the high dose group. **B**. Enrichment distribution of the overrepresented terms in the high dose group. **C**. Network of interacting genes in the top three enriched GO terms

The host gene expression within cells and tissues was linked using the pig and human gene atlases. The transcriptome profiles were primarily associated with immunity, consistent with the upregulation of genes in monocytes, macrophages and lymphocytes. In the high dose group, only three molecular mechanisms were significantly enriched for transmembrane signaling receptor activity and molecular transducer activity (Fig. 5A and B). Interestingly, the genes represented here were downregulated (Fig. 5C), including signal transduction and transcription regulatory molecules shown in Supplementary Table 3. The cytokine regulators and signal transduction molecules are critical in stimulating apoptosis and inflammation [26, 37].

**Fig. 6:**
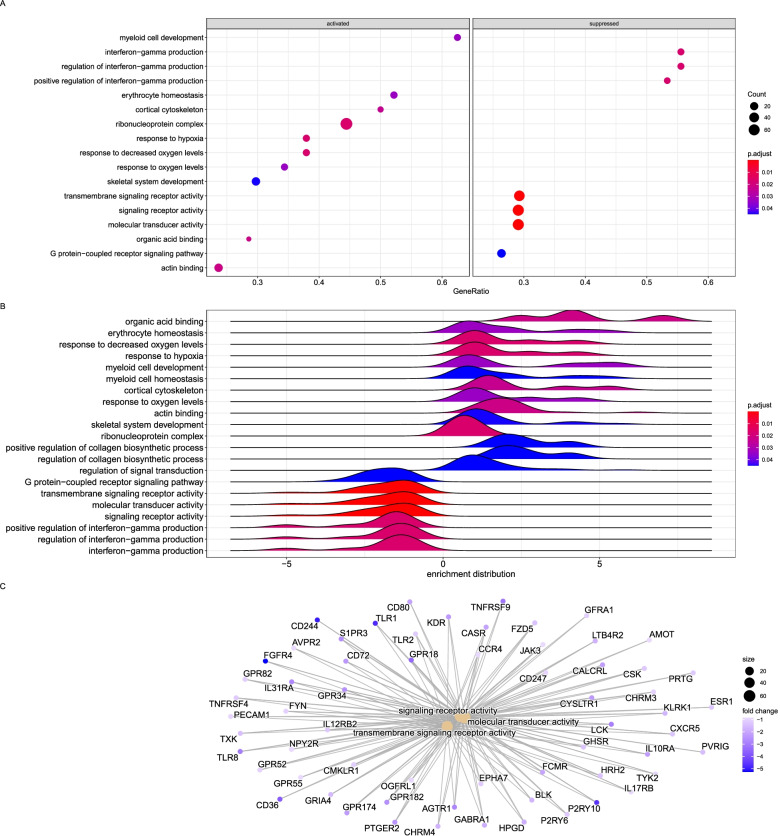
Top enriched KEGG pathways and GO annotations of the common DEGs identified from the medium dose group. **A**. Overrepresented gene ratios of significantly expressed genes activated or suppressed in the medium dose group. **B**. Enrichment distribution of the overrepresented terms in the medium dose group. **C**. Network of interacting genes in the top three enriched GO terms color-coded by their fold change value

The enriched terms were immune responses to infection and biotic stimulus in the medium dose group (Fig. 6). The genes represented by these terms were predominantly upregulated, with a few being downregulated (Fig. 6C). The upregulated genes for the immune response include *LTF, CCL3L, TNFS13, IL6,* and *TGFB3*, all over 3-fold, while *CD244* was downregulated by 4.7-fold (Fig. 6C).

**Fig. 7:**
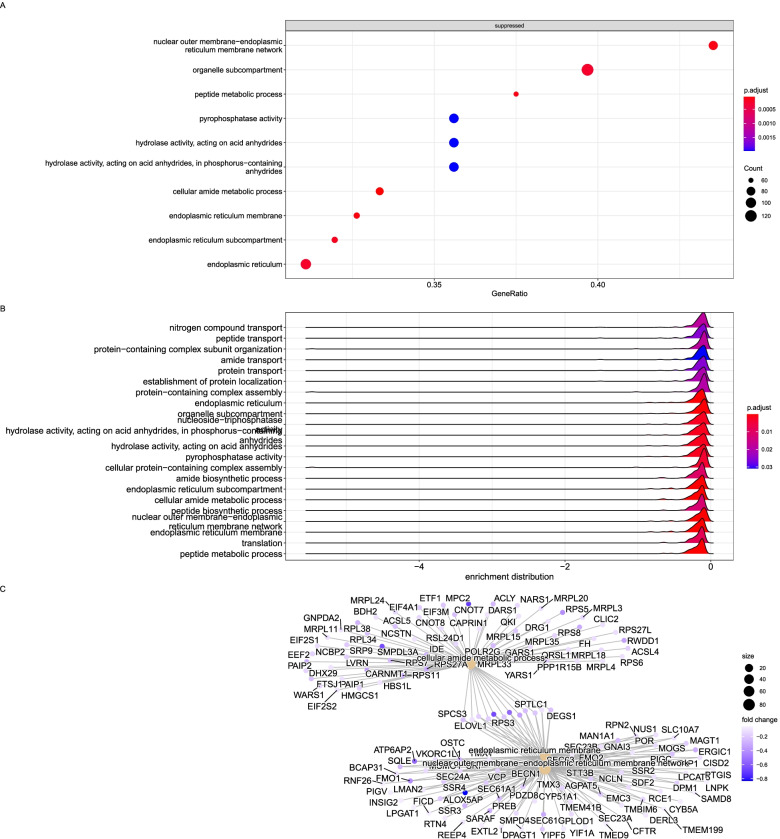
Top enriched KEGG pathways and GO annotations of the common DEGs identified from the low dose group. **A**. Overrepresented gene ratios of significantly expressed genes activated or suppressed in the low dose group. **B**. Enrichment distribution of the overrepresented terms in the low dose group. **C**. Network of interacting genes in the top three enriched GO terms color-coded by their fold change value

Another significantly activated gene in the low dose group was CD164 (endolyn), which encodes a transmembrane sialomucin. This cell adhesion molecule regulates the proliferation, adhesion, and migration of hematopoietic progenitor cells and is also a significant contributor to tumorigenesis in normal human cells [80]. The encoded protein by CD164 also interacts with the C-X-C chemokine receptor type 4 and may regulate muscle development [105]. CD164 is enhanced by the FOXK2 gene (also known as interleukin enhancer-binding Factor 1) transcriptional regulator involved in glucose metabolism, aerobic glycolysis, and autophagy. When all GO terms were considered, we detected suppression of a set of genes involved in protein translation, phosphatase activity, and replication peptide metabolic process (Fig. 7).

## Discussion

ASFV infections result in varying clinical outcomes depending on the virulence of the isolate but also the amount of infective dose used [45, 106]. As in other viral infections, the host responds by triggering biological processes, generally through gene expression, to counteract the effect of invading pathogens, including apoptosis, autophagy, and stress-induced unfolded protein response (UPR), inhibiting ASFV virus replication. Several theories exist to elucidate the molecular mechanisms of the differences in ASFV pathogenesis, such as hemorrhagic fever, top among which is the cytokine storm triggered by overexpression of proinflammatory cytokines by monocytes and macrophages, typical of ASFV infections [23, 26]. Our study used a virulent ASFV isolate from Kenya to infect locally-adapted pigs (anecdotally reported to tolerate ASF) at varying doses (high, medium, and low) to produce an effective dose that mimics severe and mild disease and determine the variations in expression levels of the genes in these locally-adapted pigs and the potential molecular mechanisms of severe and mild ASF disease.

Our results confirm that the amount of infective material determines the clinical outcomes ranging from mild clinical signs (in the low dose group of pigs) to acute hemorrhagic fever and death (as in the medium and high dose groups). The pigs in the high and medium dose groups showed severe symptoms of ASF from 6 dpi and were humanely euthanized from 7 to 17 dpi (Fig. 1). Conversely, the pigs in the low dose group did not develop severe disease and survived to the end of the experiment (29 dpi). We determined the ASFV dose to induce lethal ASF disease as 10^4^ HAD_50/ml,_ while a low dose of 10^2^ HAD_50/ml_ mimicked exposure to ASF and the presence of the virus in tissues as reported in field situations [7, 8, 28]. The limitation of this study is in the wide variations in the number of infected cells arising from differences in the time of euthanasia and thus differences in sample collection time points for the three different study groups ranging from 7 to 29 dpi.

The study reports the transcriptomic profile of spleen tissues following an experimental challenge with three different doses of ASFV. The spleen filters blood containing abnormal cells and pathogens and facilitates potential interactions between antigen-presenting cells (APCs) and cognate lymphocytes [48, 107]. The spleen allows the analysis of changes in gene expression in a mixed population of lymphatic system cells yielding a snapshot of the interactions between the host cells and pathogens. We identified several biological functions and pathways associated with response to viral infection, immune response, protein, and carbohydrate metabolism at varying levels between the low, medium, and high dose groups. Notably, in the surviving low dose group, the significant KEGG pathways consisted of highly downregulated genes (*FGA, FGB* and *FGG*) which are implicated in inflammation and thrombosis [108], indicating that survival entailed significant suppression of these pathophysiological processes.

Interferons (IFNs) form part of the first line of defense against viral infections and play a substantial role in the early immune response cells [109]. IFNs provide host defense against viral infections by inducing the expression of numerous IFN-stimulated genes (ISGs), activating host antiviral immunity [26, 110]. We detected significant upregulation of TNF inflammatory cytokines (Table 3). More TNF transcripts were detected in the medium and high dose groups, indicating that TNF plays a role in disease severity and that ASFV regulates the immune response by manipulating cytokine expression in host cells [109]. Prostaglandin E (PGE2), which modulates the immune system by regulating the expression of cytokines [87], was upregulated by over 4-fold in the medium and high dose groups.

There was significant upregulation of interferon-inducible transmembrane (IFITM) proteins. IFITMs are ISG products that confer cellular resistance to several viruses [111–114]. IFITMs are thought to function by either (i) blocking viral entry and release from cells by altering membrane rigidity and curvature, thus suppressing viral membrane fusion [111, 112, 115, 116] or (ii) by inhibiting viral replication mechanisms [111]. *IFITM1*–*3* are expressed by T cells and regulate CD4^+^ T helper cell differentiation in a T-cell-intrinsic manner affirming their role in adaptive immunity. For example, IFITM3 was shown to play a critical role in the early phase of viral entry during influenza A infection by clustering on virus-containing endosomes and lysosomes a couple of hours after infection [117, 118]. Given that ASFV enters into the host macrophages by dynamin-dependent and clathrin-mediated endocytosis and macropinocytosis [119], the upregulation of *IFITM2* and 3 alters the distribution of early and late lysosomes and endosomes in the absence of *IFITM1* during ASFV infection [120]. *IFITM1* and *IFITM3* genes were significantly upregulated in the medium dose group, indicating the alteration of the endosomal compartment in an attempt to inhibit virus entry.

ASFV infection causes neutrophilia in pigs: a higher neutrophil count in the blood than the normal reference range typical during infections and inflammation [121]. We detected upregulated expression of colony-stimulating factor 3 receptor (*CSF3*), also called the receptor for granulocyte colony-stimulating factor (*G-CSF*), that contributes to the proliferation and granulocyte differentiation of myeloid progenitor cells [122]. *CSF3* or *G-CSF* is a neutrophil growth-promoting cytokine that was significantly upregulated in the medium and high dose groups. The neutrophil recruiting chemokine, *CXCL2* [123], was significantly upregulated in the medium and high dose groups. The other chemokines detected in this study (Table 4) involved in monocyte, T and NK cell recruitment were *CCL2, CCL4* and *CCL23*, which were upregulated in the medium and high dose groups, resulting in an increased number of cells susceptible to infection [124]. The expression of CCL21 and CCL26, which have a role in the recruitment of CD8^+^ effector T cells and Th2 T lymphocytes, was suppressed, while the expression of chemokines involved in neutrophil trafficking was induced (Table 4). Induction of *CXCL10* may result in lymphocyte priming toward the Th1 phenotype or induction of T lymphocyte apoptosis. Interestingly, the *CXCL10* was significantly downregulated in the surviving low dose group indicating suppression of neutrophilia. These results corroborate the reports that ASFV-infected pigs develop neutrophilia, a critical antiviral response [26, 45].

*IL-17B* is expressed in neutrophils, germinal center B cells, neurons, stromal cells, and gut epithelium [125]. The upregulation of cytokines of the IL-17 family mediates signaling through the ACT1-dependent pathway, causing activation of pro-inflammatory factors, for example, nuclear factor (NF)-κB, associated with innate immune signaling [125]. Reports show that IL-17B promoted the TNF-α-induced production of *G-CSF* and *IL-6* in the fibroblasts and induced the expression of inflammatory cytokines of IL-1α, IL-6, and IL-23. Sun et al. showed that intraperitoneal injection of rhIL-17B caused indirect recruitment of neutrophils to the peritoneal cavity and induced the migration of CXCR4^+^ or CXCR5+ GC B cells due to *CXCL12* and *CXCL13* [126]. We detected upregulation of expression of *IL-17B* in the medium and high dose groups, while *CXCL12* was downregulated, contributing to our understanding of the ASFV pathogenesis.

ASFV infection has been shown to upregulate the expression of immunosuppressive cytokines to put a strain on the host immune response. Previous studies have shown that *IL27*, a suppressor of the immune response of Th1 and Th17 cells, is downregulated during ASFV infection [127]. However, *IL27* was significantly upregulated in our study by over 2-fold in the medium and high dose groups. Interleukin 1 receptor antagonist (*IL1RN*), an endogenous pyrogen, causes an inflammatory response and promotes B cell proliferation and differentiation when combined with IL-1R [128]. IL-1R was upregulated in the medium and high dose groups, which may result in the exacerbated inflammatory response in these two groups. We detected the high expression of the ASFV L83L gene in the high and medium dose groups but only lowly (in TPM) in the low dose group. The ASFV L83L gene encodes a protein that interacts with the host IL-1β and its deletion did not affect IL-1 production, indicating that the host could be using an alternative mechanism for IL-1 production [129]. The interleukin-1 receptor-associated kinases (IRAKs) are crucial mediators of toll-like receptor and interleukin-1 receptor signaling processes [94]. The expression of IRAKs was detected only in the high dose group, where IRAK4 and IRAK3 were suppressed while the expression of IRAK1 was upregulated. Recent studies have shown that the actions of IRAK-1 participate in the stimulation of NF-kappaB-dependent transcriptional events [130].

It has been shown previously that the variation between the ASFV genomes of different isolates is primarily a result of the increase (gain) or decrease (loss) of members of the multigene families, namely *MGF-100, MGF-110, MGF-300, MGF-360* and *MGF-505/530* families [131, 132]. Our study detected, on average, 172 ASFV genes and multigene families (Supplementary Table 4 and 5) with higher gene count in TPM detected in the *MGF 100-1 L, E184L,* and *B646L* genes. The MGF 360 has been shown to have a high gene copy number and is highly variable, containing 22 paralogous genes in total [16]. We did not detect a critical gene, DP71L, which codes for a protein that functions as a cofactor for the pig host protein phosphatase 1 (*PP1*), a phosphatase that dephosphorylates *P-eIF2α* [62, 86]. However, upregulation of the host *PPP1R15A* explains why the loss of DP71L does not affect host cell translation during ASFV infection. *PPP1R15A* codes for the *GADD34* complex, a host protein that dephosphorylates P-eIF2α by an interferon-induced double-stranded RNA-activated protein kinase (PKR) in a prominent cellular antiviral pathway [62, 86]. Since *PPP1R15A* was significantly upregulated in high and medium dose groups, it likely provided a mechanism to ensure *P-eIF2α* is maintained in a dephosphorylated state during ASFV infection [45]. Following infection, *P-eIF2α* initiates the transcription of *ATF4*, which then upregulates the expression of UPR-associated genes, including *PPP1R15A* and an inducer of apoptosis called *CHOP*. Our study detected upregulation of *ATF4* in the medium and high dose groups, which further affirms the apoptosis in ASFV pathogenesis.

ASFV, like other viruses, can manipulate immune responses to promote infection, such as the delayed onset of apoptosis and autophagy, by expressing several genes that evade the host response [133, 134]. The highly conserved and immunodominant ASFV proteins, namely *E184L, CP312R, K205R,* and *K145R* [45], were significantly upregulated in the high and medium dose group. Autophagy and apoptosis have a critical role in the innate and adaptive immune response against viral infection [135] and in delivering antigens for MHC Class II epitope processing [136]. Our data indicate that host gene expression following ASFV infection could suppress apoptosis, particularly autophagy-associated apoptosis. Up-regulated genes overrepresented the apoptosis processes, and there was a significant overrepresentation of down-regulated genes involved in autophagy. *NUPR1* suppresses metabolic stress-induced autophagy-associated programmed cell death by apoptosis and programmed necrosis [64] and was significantly up-regulated in the high dose group 5-fold. We also detected the upregulation of a pro-apoptosis and an autophagy inducer gene called BCL2 interacting protein 3 (*BNIP3*), further confirming that apoptosis and autophagy were enhanced in the high and medium dose groups. Further, *GADD45B* is known to suppress apoptosis and autophagy and it was significantly upregulated in the high and medium dose groups. Another crucial regulatory gene in the UPR pathway, *PPP1R15A*, was significantly upregulated after ASFV infection. Other signaling transducers were downregulated in the high and medium dose groups, resulting in delayed apoptosis induction by high TNF expression.

Autophagy-related genes such as *GAS2* and *GAS2L3* were downregulated, contrasting observations in ex vivo macrophages [26]. We also found the suppression of expression of one autophagy-related gene called *FUNDC1* in the low dose group. *FUNDC1* is a crucial mitochondrial outer-membrane protein that is a receptor for hypoxia-induced mitophagy [137]. Hypoxia-induced mitophagy is a specialized process that occurs in hypoxic cells whereby the dysfunctional mitochondria are selectively removed [138]. Thus, suppression of *FUNDC1* results in a defect in hypoxia-induced mitophagy. FUNDC1 has been shown to work with *DRP1* and calnexin [*CALN*] (suppressed at 1.36-fold in the high dose group) during hypoxic conditions in mammalian cells [138]. Thus, detection of suppression of *FUNDC1* in low dose groups confirms that the pigs in this group likely survived the ASFV infection by suppressing hypoxia-induced mitophagy.

Signal transducers and transcription factor genes are essential in M1 macrophage polarization. Expression of *TAB3* and the other five critical immune transcription factors, *FOSB, FOS, IRF7, JUNB, IRF4,* and *IRF8*, were all upregulated apart from IRF4 and IRF8, which were downregulated in the high and medium dose groups (Supplementary Table 3). *FOS* and *JUN* are early response genes whose expression is induced by cell-extrinsic and cell-intrinsic signals like during viral infection [139]. Contrary to previous reports [26], our study showed that *JUN* and *FOS* were upregulated in the medium and high dose groups.

Cytokines, growth factors, and hormones utilize the Janus kinase–signal transducer and activator of transcription (JAK-STAT) pathway to transmit their information into the cell nucleus. At the same time, the cytokine signaling inhibitors such as ubiquitin-specific peptidases (USP) and Suppressors of Cytokine Signaling (SOCS) downregulate interferon responses. SOCS protein family inhibits STAT activation by many JAK-STAT activating receptors [140]. Our results corroborate further that ASFV immune evasion could be via interference with the NF-κB, JAK, and IRF signaling pathways.

ASFV encodes genes that allow it to evade the host defense systems and modulate host cell function. *A224L* is an ASFV gene that inhibits apoptosis, which is vital in host-pathogen interaction [21]. Though not differentially expressed, we detected more *A224L* transcripts in the high and medium dose groups (172 and 303 TPM, respectively) compared to the low dose groups (13 TPM), indicating that the virus was attempting to evade the apoptosis process, especially at a higher viral dose. The expression of ASFV *A238L* gene was suppressed in the high and medium dose groups compared to the surviving low dose group. The ASFV gene, *A238L*, is a known inhibitor of host gene transcription through inhibition of NF-kB and calcineurin phosphatase [141, 142] that has been frequently detected in surviving pigs and modulates the host immune responses [92, 143, 144], as was the case in this study. In the pig host, the RELA proto-oncogene [NF-kB subunit, or p65 (*RELA*)] was detected at higher counts (2846 TPM) in the low dose group than the high and medium doses at 257 and 269 TPM, respectively (Supplementary Table 2). RELA has been associated with resistance/tolerance to ASF [25], and the fact that it was downregulated in the high dose (1.94-fold) and medium dose (2.34-fold) confirms the lethality of this dose in these reportedly resilient pigs.

The ASFV CD2v (*EP153R*) activates NF-κB, subsequently inducing IFN signaling and apoptosis in swine macrophages [110]. The *EP153R* gene is vital in the haemadsorption of red blood cells and suppresses the expression of MHC class I molecules by impairing the exocytosis process without affecting the synthesis of MHC antigens [145]. Additionally, proteases found on macrophages called Cathepsins process antigens before loading on MHC class II molecules [146]. We determined that antigen processing and presenting cells were downregulated in the medium and high dose groups. Cathepsin S (*CTSS*) was downregulated in both the medium and high dose groups (Table 5). The swine *SLA-DMA* and *SLA-DMB* are required in epitope loading onto MHC Class II molecules by removing the invariant chain in the groove of the MHC molecules, while *SLA-DOA* and *SLA-DOB* inhibit this process [147]. The *SLA-DMB, SLA-DQA, SLA-DRA, SLA-DRB,* and *SLA-DOB* were down-regulated in the medium and high dose groups in contrast to a previous study where *SLA-DOA* and *SLA-DOB* were up-regulated following ASFV infection [26]. The MHC class II genes were downregulated in the medium and high dose group of pigs, indicating that the MHC class II activity is compromised during ASFV infection. The TNF-like weak inducer of apoptosis (*TWEAK*) is a member of the TNFSF ligands [148]. *TNFSF9* (4-1BBL/CD137) and *TNFRSF11A* were downregulated in the high and medium dose groups. *TNFSF9* contributes to the clonal expansion, survival, and development of T cells induces proliferation in peripheral monocytes and enhances T cell apoptosis induced by TCR/CD3 triggered activation [149]. *TNFRSF11A* also regulates CD28 co-stimulation to promote Th1 cell responses. Additionally, TRAF adaptor proteins have been shown to bind to this receptor and transduce the signals leading to activation of NF-kB. On the other hand, *TNFSF11* are important regulators of interactions between T cells and dendritic cells [150]. In this regard, ASFV infection inhibits MHC class II antigen presentation and retards T cells activation by antigen-presenting cells such as macrophages [26].

Overall, our study provides the first gene expression data from locally-adapted Kenyan pigs following experimental infection with a highly virulent ASFV genotype IX isolate, Ken/busia.1 (Ken-1033), at varying doses to mimic acute and mild disease. Our results indicate a strong correlation between severe ASF pathogenesis in the high and medium dose groups with upregulation of proinflammatory TNF cytokines, *IL17B* and *IL6*, while expression of *IFN-ω*, *IL16, IL10RA, IL21R*, *TNFRSF9, TNFRSF11A,* and *TNFRSF21* was suppressed. We showed that the ASFV pathogen increases its replication and transcription machinery to evade the host immune response, while the pig host induces immunological and stress response pathways to counter the viral attack. Additionally, ASFV infection suppressed genes involved in MHC antigen processing and presentation by down-regulation of Cathepsins S (*CTSS*), *SLA-DQB1, SLA-DOB, SLA-DMB, SLA-DRA, and SLA-DRA SLA-DQA, TNFSF11* and *TNFSF13*, and up-regulation of *SLA-DOA* and *SLA-DOB*. Conversely, we detected suppression of the critical genes involved in inflammation and neutrophilia in the surviving low dose groups. However, the spleen samples sequenced in this study comprised a mixed population of infected and uninfected bystander cells and thus, the changes in host gene expression are also likely attributed to a bystander effect rather than a direct effect on the infected cells alone [151].

## Conclusions

Our study corroborates most previously available data on host responses and adds to the growing body of knowledge about the cytokines, chemokines, and regulatory factors involved in local and systemic ASFV pathogenesis and immune evasion. Our study shows that the locally-adapted pigs induced expression of protective genes associated with tolerance to infection and repression of genes involved in inflammation at varying levels. Further, the array of differentially expressed genes detected augments our knowledge of ASF pathogenesis critical in understanding the intricate host-pathogen interaction, especially during exposure to varying ASFV doses as in field situations. The survival of the low dose group strongly indicates the high likelihood that the local pig breeds are tolerant to ASFV at a 10^2^HAD_50_ low dose. However, these pigs may serve as carriers and sources of acute new infections, further contributing to the persistence of ASFV in swine populations [152]. We recommend further studies that compare ASF pathogenesis in response to low doses of ASFV between locally-adapted pigs and exotic pig breeds to underpin genetic variations, if any.

## Materials and method

### Ethics statement

All animal experiments reported in this study were approved by Institutional Animal Care and Use Committee (IACUC) and Institutional Biosafety Committee (IBC) of the International Livestock Research Institute (ILRI), Kenya (Reference: VN_IACUC-2011-04). All methods were carried out in accordance with the approved protocol and relevant regulations. All the pigs were maintained, sampled, and euthanized humanely by the institutional veterinarian. All methods are reported in accordance with ARRIVE guidelines for the reporting of animal experiments.

### Experimental design, study animals and viral isolate

A total of 14 locally-adapted pigs were used in this study in a biosecurity level II animal facility at ILRI, Nairobi. The pigs were 6-months old African breeds (also termed herein as locally-adapted) from Homa Bay County in South-western Kenya of both genders (male and female); purchased from local farmers who reared them in a free-range management system. The pigs were transported to the ILRI Nairobi farm facility, quarantined and acclimatized for 21 days.

The ASFV virus was isolated from a spleen tissue detected in Sagalame, Busia County, Kenya, and has been extensively characterized and determined to be genotype IX [GenBank ID: KM000146.1] [49]. The spleen tissue was homogenized and passaged in peripheral blood monocytic cells (PBMCs) to obtain a pure isolate of the Ken12/Busia.1 ASFV. The culture supernatant was freeze-thawed severally to lyse the cells and expose the virus. The virus was diluted ten-fold to the required doses used in the experiment. The dilutions were based on an end-point virus titration of the original material on macrophages derived from PBMCs. Before the ASFV challenge, blood samples were collected from all the studied pigs and ASFV diagnostics targeting the conserved VP72 capsid protein-coding region of the ASFV genome were conducted by qPCR [153]. A negative qPCR result qualified the pigs for inclusion in the experiment.

After a 21-day acclimatization period, the pigs were moved into a BSL2 facility at ILRI Nairobi and randomly assigned in the respective groups then inoculated intramuscularly with 1 ml of the ASFV Ken12/busia.1 isolate as follows: uninfected control pig (sterile PBS; *n* = 1; pig #1); an experimental low dose (10^2^HAD_50_/ml) ASFV infection group (*n* = 3; pigs #2, #3 and #4); an experimental medium dose (10^4^HAD_50_/ml) ASFV infection group (*n* = 5; pigs #5, #6, #7, #8 and #9); and experimental high dose (10^6^HAD_50_/ml) ASFV infection group (*n* = 5; pigs #10, #11, #12, #13 and #14). While the intramuscular (IM) route does not represent the natural infection, it is reported to be the most reliable route of challenge, allowing high infection incidence, allowing more control of infective dose and timing of challenge [21, 154]. Back titration was carried out to confirm the administered dose. All the pigs were housed in a separate room based on infective dosage and gender, isolated from the uninfected control pig to avoid contact and, consequently, ASFV transmission.

The pigs were monitored daily for symptoms of the disease, and their body temperatures were recorded. Pig blood was collected every other day from the jugular vein using BD Vacutainer® needles gauge 20 (Becton, Dickinson and Company, New Jersey, USA) into 10 ml BD Vacutainer® glass serum tubes and 10 ml 15% EDTA tubes. Whole blood in heparin was harvested per pig for haemadsorption assays, whole blood in EDTA from each pig for use in virus detection and infection monitoring by qPCR, and 5 mL of whole blood to harvest serum. All the samples were well labeled and transported in a cool box to the laboratory for storage at − 80 °C and further processing. Back titration of the viral innocula showed that the pigs received the required dose per group. The pigs that reached a humane end-point were anesthetized by intramuscular injection with Xylazine + Ketamine (Merck KGaA, Darmstadt, Germany) at a dose of 2 mg/kg + 25 mg/kg. Sedated animals were humanely euthanased using Sodium pentobarbital 390 mg + Sodium phenytoin 50 mg/ml (Euthasol®, Le Vet. Pharma, The Netherlands) injected intravenous into the jugular vein at a dose of 0.22 ml/kg. A post-mortem was carried out, and all organs and tissue samples were collected and stored in 50 ml Falcon® tubes at − 80 °C until further analyses.

### Total RNA isolation, library construction, and sequencing

Total RNA was isolated from 10 porcine spleen samples (3 each for the high, medium and low dose groups, and one uninfected control). Total RNA was extracted from 10 mg of spleen tissue using a modified protocol described previously [155] that combines tissue lysis with TRIzol™ (Thermo Fisher Scientific, USA) and silica-column purification using the RNeasy® Mini Kit (Qiagen, Germany) with on-column DNase I treatment step (Thermo Fisher Scientific, USA). The pure RNA was eluted from the columns using 40 μl of nuclease-free water. The RNA quality was assessed with the 2100 Bioanalyzer and Eukaryote Total RNA Nano Kit (Agilent Technologies, Inc., CA, USA). The total RNA quantity was determined using an ssRNA assay on the Qubit® 2.0 fluorometer (Thermo Fisher Scientific, USA). The total RNA purity was determined on a NanoDrop™ spectrophotometer (Thermo Fisher Scientific, USA), considering that the absorbance ratio at 260 nm and 280 nm close to 2.0 indicates highly pure RNA. The stranded RNA-Seq libraries were prepared with the KAPA RNA HyperPrep Kit with RiboErase (Roche, USA) to deplete the rRNA following the manufacturer’s instructions. Each stranded library was paired-end sequenced (2 × 150 bp) in an Illumina NovaSeq 6000 S4 platform at Quick Biology Inc. (Pasadena, CA, USA).

### Statistical and bioinformatic analyses

The quality of the paired-end reads generated from the Illumina NovaSeq 6000 System was checked using FastQC v 0.11.7 [156]. The sequencing adapters and low-quality reads were removed using Trimmomatic v 0.38 [157] using a sliding window approach and trimming the reads once the average quality within the window fell below the given threshold. We removed duplicates in BBMap v38.67 (Bushnell, 2014). The preprocessed reads were simultaneously mapped to the pig host (*Sus scrofa* build 11.1) and ASFV pathogen (Ken06.Bus; Genbank ID KM111295.1) reference genomes [158] using STAR v 2.5.3a [159]. The resulting alignments were used to generate gene counts using featureCounts, part of the Subread v1.6.2 suite [160]. Differential gene expression analysis was performed using DESeq2 [161] in R v.3.6 [162]. To obtain the gene counts in transcripts per million, we transformed the raw counts by normalizing them based on gene lengths and sequencing depth.

Data exploration using principal component analysis allowed us to detect and remove outlier samples from downstream analysis. Lowly expressed genes, those with a low number of gene counts per sample, were also removed at a threshold of 100 gene counts. The count data were transformed by the varianceStabilizingTransformation function in DESeq2 [161], which normalizes the raw counts using size factors. A list of significant and differentially expressed genes was detected for each set using the Benjamini-Hochberg (B.H.) multiple testing procedure on the *p*-values to obtain adjusted *p*-values (threshold 0.05). The log2 fold change was used to distinguish between downregulated and upregulated genes and the resulting genes were visualized using the Bioconductor package EnhancedVolcano [163]. To identify and visualize differentially expressed genes with overlaps in the different conditions, we used VennDiagram [164]. Genes showing differential expression were identified using ‘Severity’ (low, medium, and high) as factors using the following contrasts: High dose vs. Control, Medium dose vs. Control, and Low dose vs. Control.

### Gene ontology (GO) enrichment and mapping to the KEGG pathways

The Database of Annotation Visualization and Integrated Discovery (DAVID) v 6.750 [165] was used to annotate the differentially expressed transcripts. The significant DEGs were mapped to the KEGG pathways, and the reported gene names were listed. The DAVID gene enrichment tool was used to detect the KEGG pathways and GO terms that were overrepresented within the top DEGs. *P*-values were calculated using the Fisher exact test based on the fraction of genes that mapped to a specific pathway compared to the number of background genes associated with the pathway.

## Supplementary Information

**Additional file 1.**

**Additional file 2.**

**Additional file 3.**

**Additional file 4.**

**Additional file 5.**

**Additional file 6.**

**Additional file 7.**

**Additional file 8.**

**Additional file 9.**

**Additional file 10.**

## Data Availability

The RNA-seq data generated in this study are accessible on the National Center for Biotechnology Information (NCBI) database under the BioProject PRJNA823664 (https://www.ncbi.nlm.nih.gov/bioproject/?term=PRJNA823664).
