# Supplementary figures and images for: Transcriptome profile of spleen tissues from locally-adapted Kenyan pigs (Sus scrofa) experimentally infected with three varying doses of a highly virulent African swine fever virus genotype IX isolate: Ken12/busia.1 (ken-1033)
Source: BMC Genomics. 2022 Jul 19;23:522. doi: 10.1186/s12864-022-08754-8 (PMC9294756; doi:10.1186/s12864-022-08754-8)

A

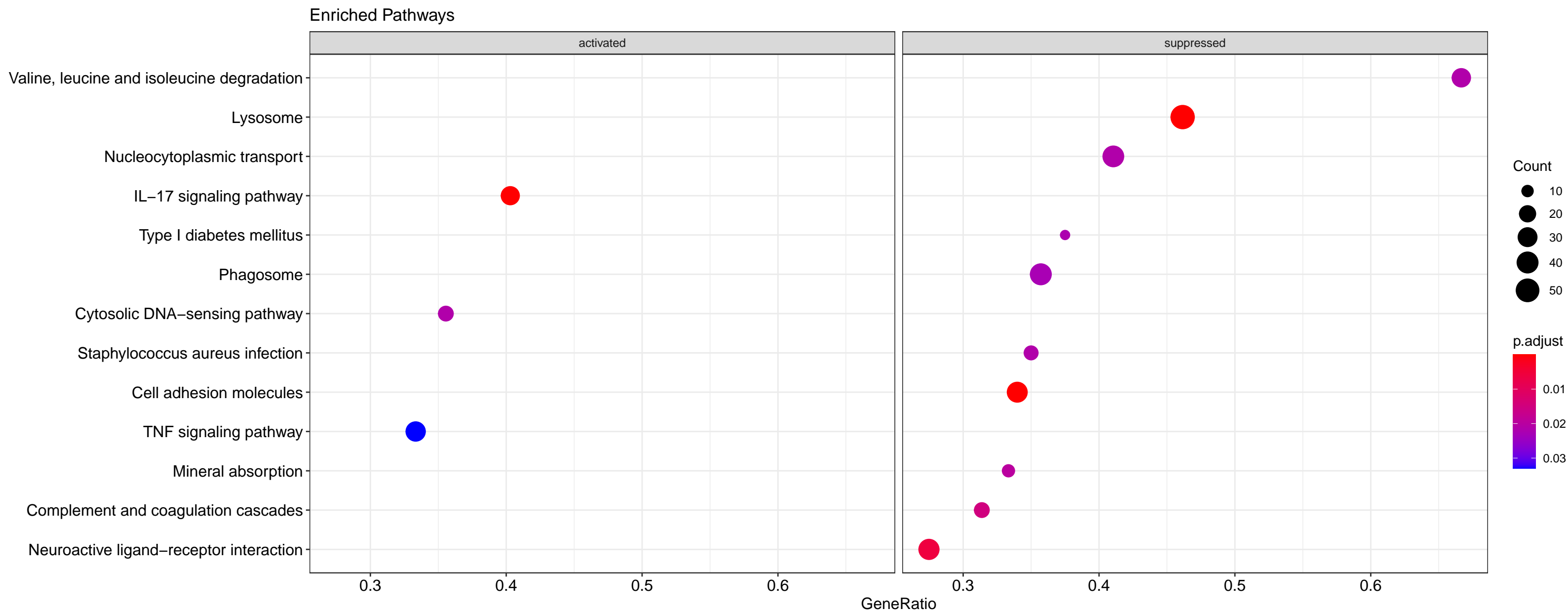

B

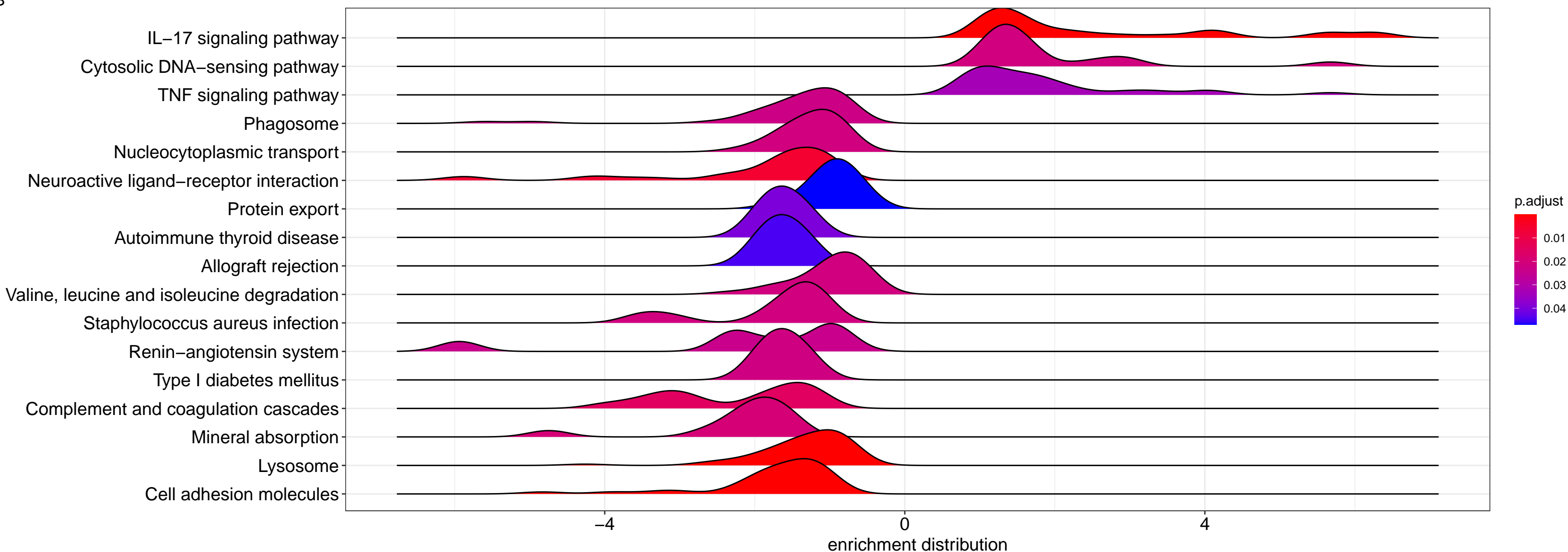

C

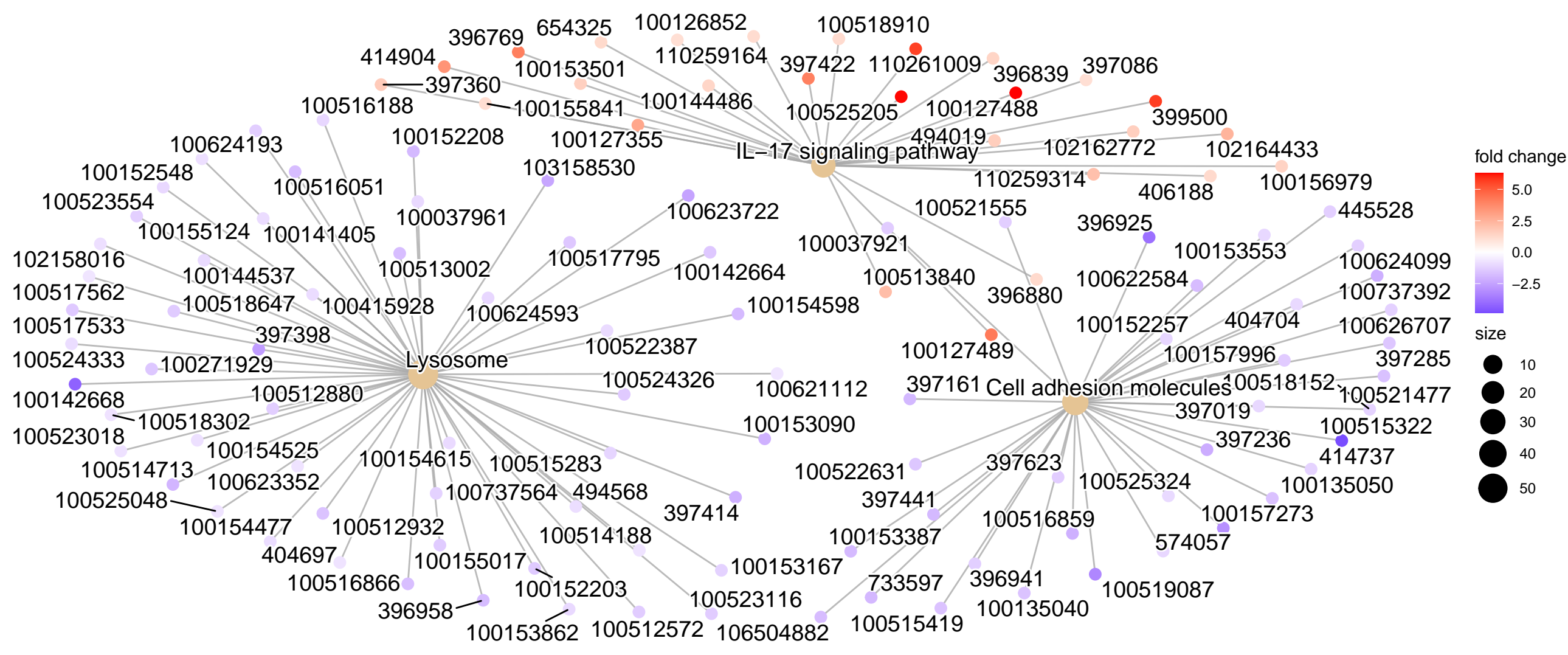

Supplement: Supplementary file 1 — Additional file 1. [file 12864_2022_8754_MOESM1_ESM.pdf]

A

Enriched Pathways

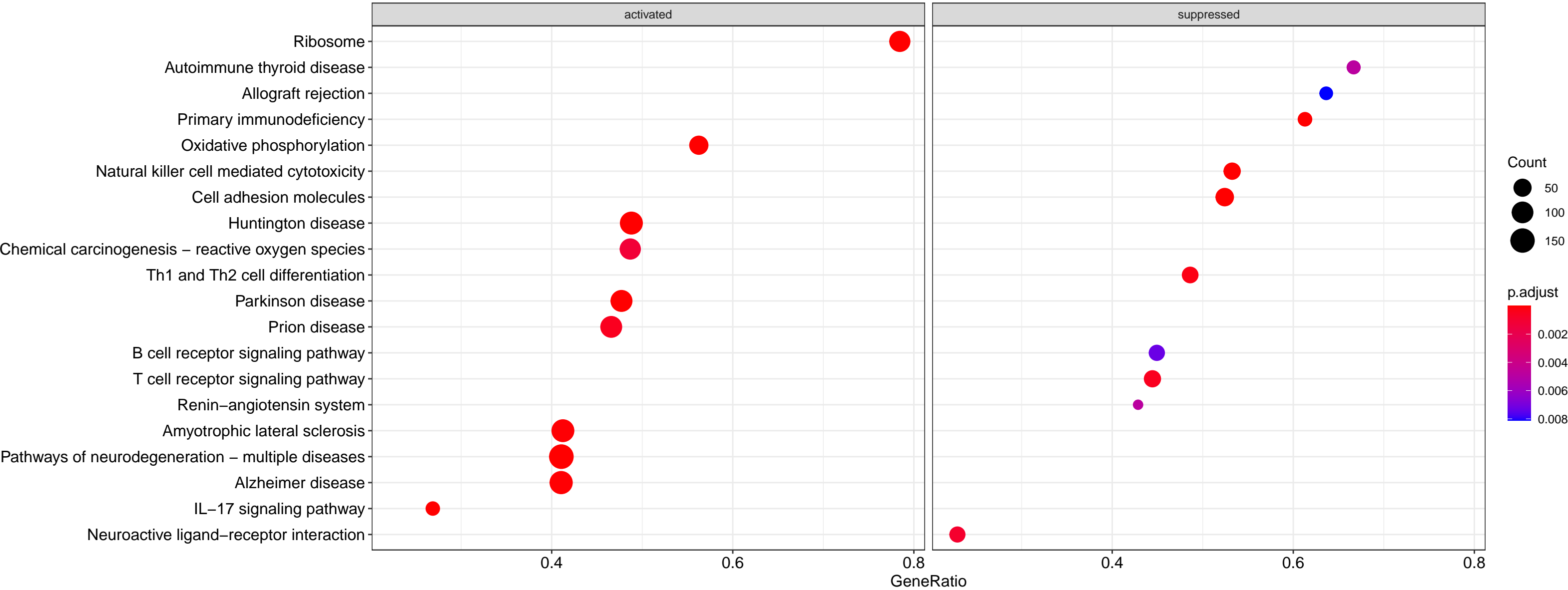

B

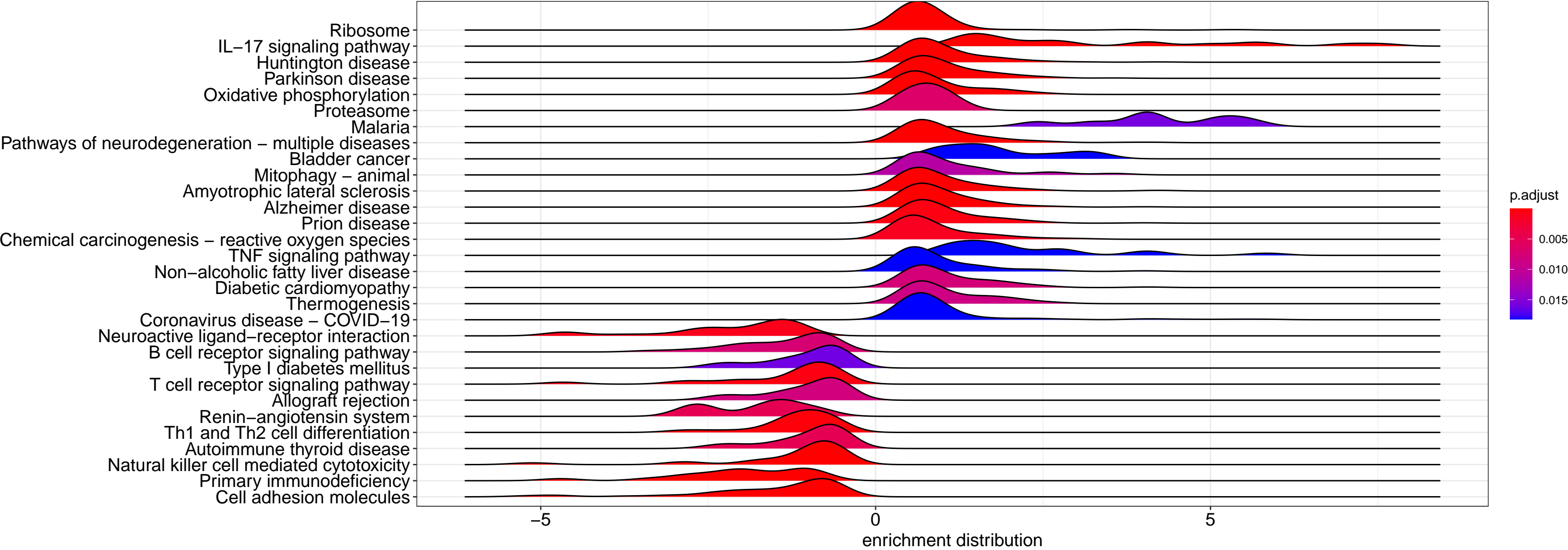

C

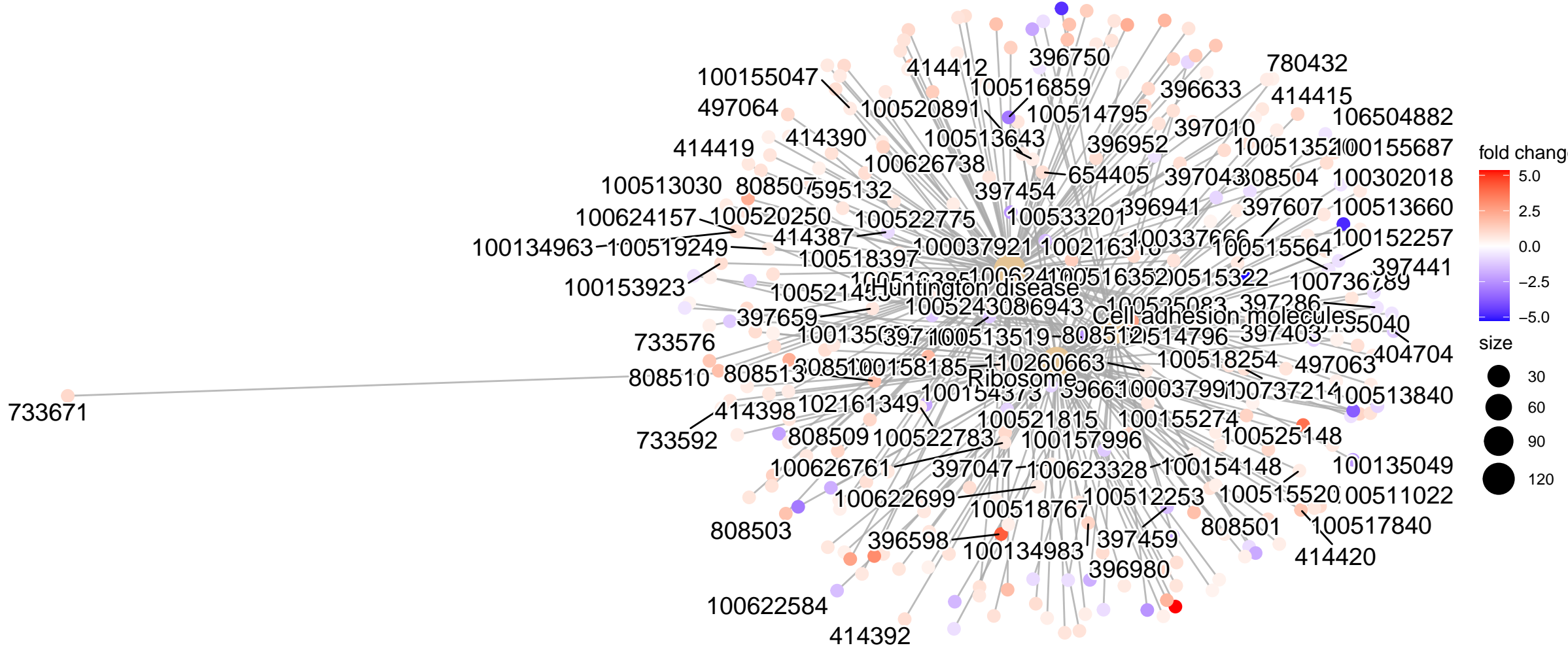

Supplement: Supplementary file 2 — Additional file 2. [file 12864_2022_8754_MOESM2_ESM.pdf]

A

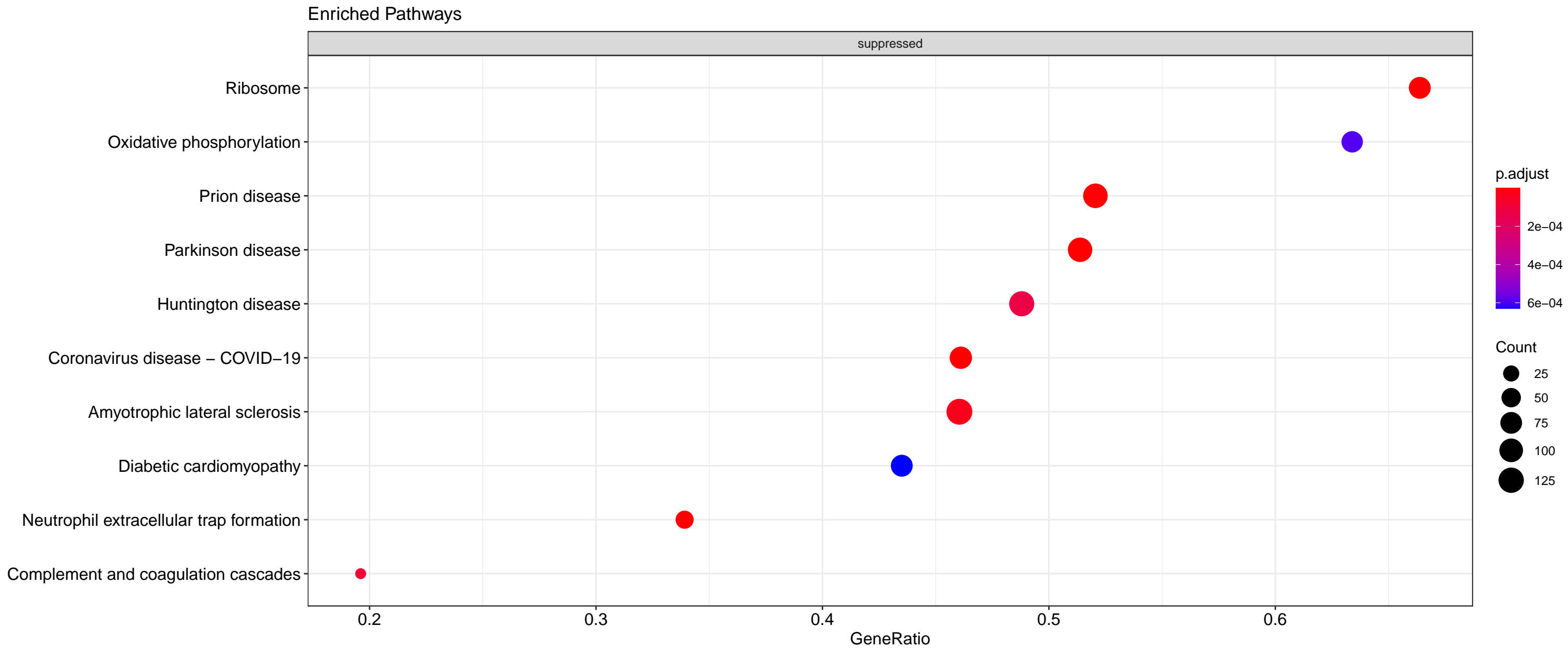

B

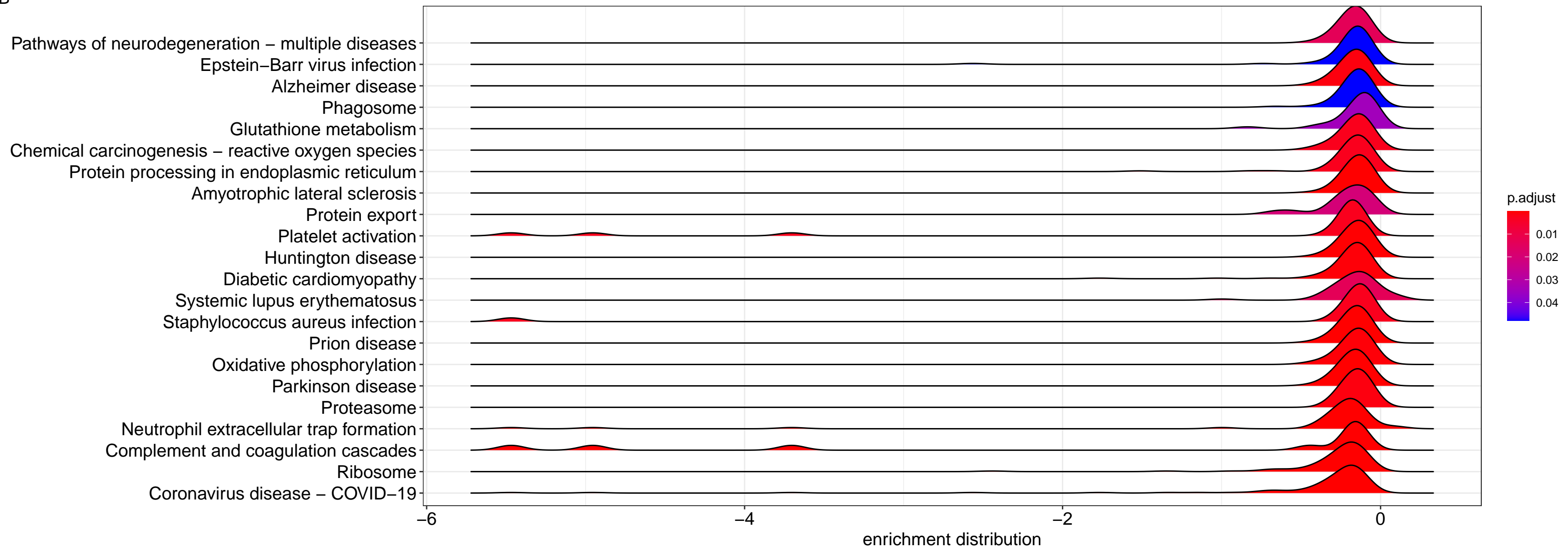

C

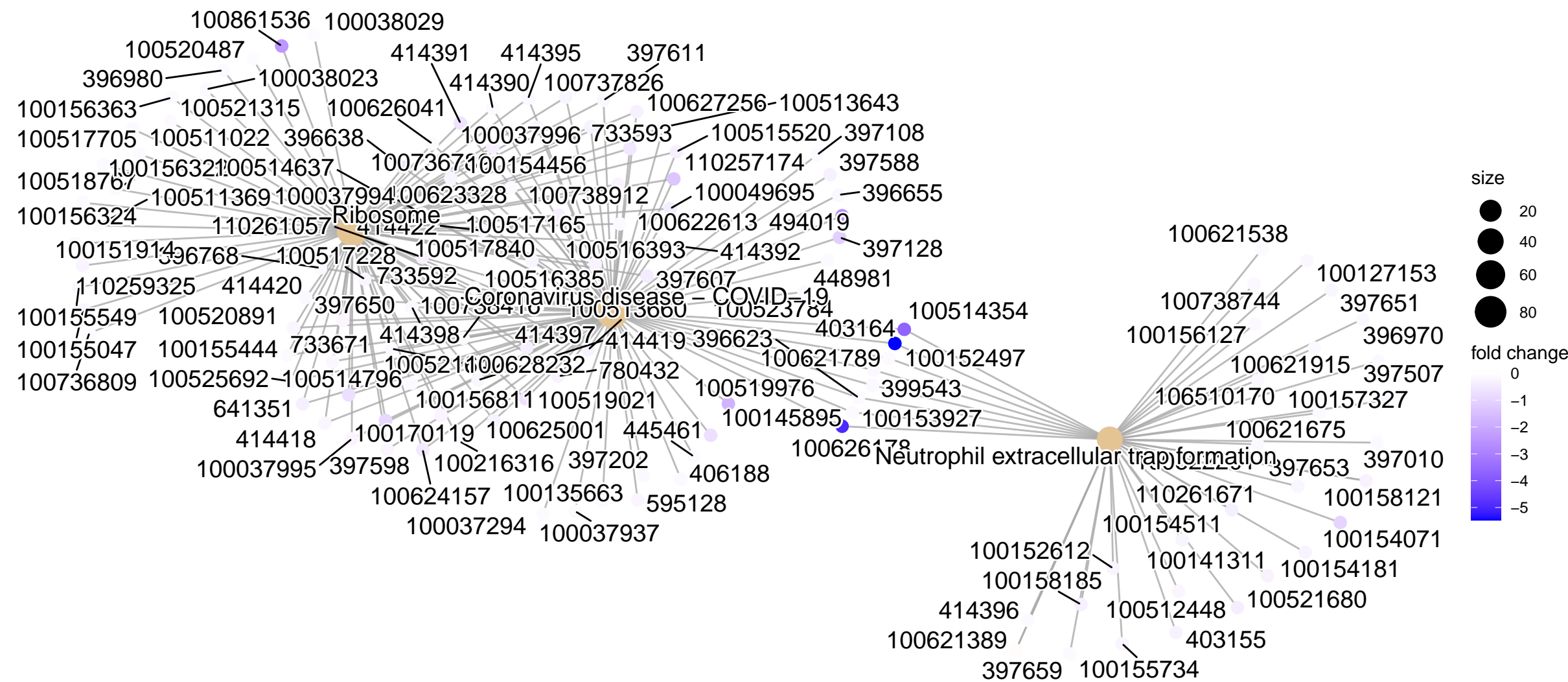

Supplement: Supplementary file 3 — Additional file 3. [file 12864_2022_8754_MOESM3_ESM.pdf]
